# A T cell receptor targeting a recurrent driver mutation in FLT3 mediates elimination of primary human acute myeloid leukemia in vivo

**DOI:** 10.1038/s43018-023-00642-8

**Published:** 2023-10-02

**Authors:** Eirini Giannakopoulou, Madeleine Lehander, Stina Virding Culleton, Weiwen Yang, Yingqian Li, Terhi Karpanen, Tetsuichi Yoshizato, Even H. Rustad, Morten Milek Nielsen, Ravi Chand Bollineni, Trung T. Tran, Marina Delic-Sarac, Thea Johanne Gjerdingen, Karolos Douvlataniotis, Maarja Laos, Muhammad Ali, Amy Hillen, Stefania Mazzi, Desmond Wai Loon Chin, Adi Mehta, Jeppe Sejerø Holm, Amalie Kai Bentzen, Marie Bill, Marieke Griffioen, Tobias Gedde-Dahl, Sören Lehmann, Sten Eirik W. Jacobsen, Petter S. Woll, Johanna Olweus

**Affiliations:** 1 Department of Cancer Immunology, Oslo University Hospital Radiumhospitalet, Oslo, Norway; 2 Institute of Clinical Medicine, University of Oslo, Oslo, Norway; 3 Department of Medicine Huddinge, Center for Hematology and Regenerative Medicine, Karolinska Institutet, Stockholm, Sweden; 4 Genomics Group, Faculty of Biosciences and Aquaculture, Nord University, Bodø, Norway; 5 Department of Immunology, Oslo University Hospital, Oslo, Norway; 6 Department of Cell and Molecular Biology, Karolinska Institutet, Stockholm, Sweden; 7 Department of Immunology, University of Oslo and Oslo University Hospital, Oslo, Norway; 8 Section for Experimental and Translational Immunology, Department of Health Technology, Technical University of Denmark, Kongens Lyngby, Denmark; 9 Department of Hematology, Aarhus University Hospital, Aarhus, Denmark; 10 Department of Hematology, Leiden University Medical Center, Leiden, the Netherlands; 11 Hematology Department, Section for Stem Cell Transplantation, Oslo University Hospital, Rikshospitalet, Clinic for Cancer Medicine, Oslo, Norway; 12 Karolinska University Hospital, Stockholm, Sweden; 13 Department of Medical Sciences, Uppsala University, Uppsala, Sweden; 14 MRC Molecular Haematology Unit, MRC Weatherall Institute of Molecular Medicine, University of Oxford, Oxford, UK

**Keywords:** T-cell receptor, Cytotoxic T cells, Immunization, Acute myeloid leukaemia, Cancer

## Abstract

Acute myeloid leukemia (AML), the most frequent leukemia in adults, is driven by recurrent somatically acquired genetic lesions in a restricted number of genes. Treatment with tyrosine kinase inhibitors has demonstrated that targeting of prevalent FMS-related receptor tyrosine kinase 3 (FLT3) gain-of-function mutations can provide significant survival benefits for patients, although the efficacy of FLT3 inhibitors in eliminating FLT3-mutated clones is variable. We identified a T cell receptor (TCR) reactive to the recurrent D835Y driver mutation in the FLT3 tyrosine kinase domain (TCR^FLT3D/Y^). TCR^FLT3D/Y^-redirected T cells selectively eliminated primary human AML cells harboring the FLT3^D835Y^ mutation in vitro and in vivo. TCR^FLT3D/Y^ cells rejected both CD34^+^ and CD34^−^ AML in mice engrafted with primary leukemia from patients, reaching minimal residual disease-negative levels, and eliminated primary CD34^+^ AML leukemia-propagating cells in vivo. Thus, T cells targeting a single shared mutation can provide efficient immunotherapy toward selective elimination of clonally involved primary AML cells in vivo.

## Abstract (web summary)

Olweus and colleagues identify a T cell receptor reactive against the recurrent D835Y driver mutation in FLT3 in acute myeloid leukemia and show that engineered T cells against this neoantigen provide efficient immunotherapy.

## Main

Neoantigens represent an attractive group of targets in cancer immunotherapy, as they are tumor specific and can be recognized by T cells as foreign in the context of major histocompatibility complex (MHC)^1–4^. Neoantigenic burden is an important determinant of clinical success upon checkpoint inhibition^5–7^, and case reports have demonstrated that neoantigen-reactive T cells can mediate clinical responses^8–10^. This has brought hope that T cells genetically modified to express TCRs derived from neoantigen-reactive T cells could provide efficient adoptive cell therapy. Patient T cells do, however, spontaneously recognize only 1–2% of candidate neoantigens predicted to be expressed and presented on human leukocyte antigen (HLA) in solid cancer^11^. As the large majority is unique to the individual patient, therapeutic targeting of neoantigens generally becomes a highly personalized effort^12^. In addition to being resource demanding, such a strategy might not benefit patients in time. By contrast, public neoantigens derived from recurrent oncogenic mutations have the advantage that a single TCR with off-the-shelf availability could target larger patient groups. Although shared mutations resulting in neoantigens presented on frequently expressed HLA alleles are rare in solid cancer, promising results were recently shown with T cells engineered to express a TCR targeting mutant KRAS in a patient with pancreatic adenocarcinoma^13^ and mutant p53 in a patient with breast cancer^14^.

AML is, in contrast to solid tumors, characterized by recurrent driver mutations in a restricted number of genes^15^, many of which are screened for in routine diagnostics. The only curative therapeutic option for many patients with AML today is allogeneic hematopoietic stem cell transplantation (allo-HSCT). Allo-HSCT remains, however, associated with high relapse rates as well as transplant-related morbidity and mortality resulting from donor T cells attacking healthy recipient cells, causing severe graft-versus-host disease^16^, and often a suitable donor cannot be identified. This has prompted development of alternative cellular therapies^17,18^. Successful targeting of the lymphoid-specific molecule CD19 in chimeric antigen receptor (CAR) T cell therapy of acute lymphocytic leukemia, has led to attempts at also directing CARs to myeloid cell surface antigens, including CD33 (ref. ^19^), CD123 (ref. ^20^) and FLT3 (ref. ^21^), overexpressed on AML cells. However, these molecules are also highly expressed on normal myeloid progenitor cells and even on hematopoietic stem cells, representing a major challenge^22–24^. Thus, CAR T cell therapies targeting these molecules are associated with toxicities and a need for transplantation to rescue normal hematopoiesis after a short period of treatment (for example, NCT03126864) (ref. ^25^). It is also unclear whether all AML-propagating cells express these antigens and therefore whether targeting has curative potential^26^.

Cytotoxic cells modified to express TCRs provide an opportunity to also target intracellular antigens with restricted expression in normal tissues, such as the specialized DNA polymerase terminal deoxynucleotidyl transferase (TdT)^27^, dramatically broadening the repertoire of potential targets. To this end, TCRs recognizing Wilms tumor 1 (WT1) were recently shown to reduce relapses in patients with AML after transplantation^28^. A TCR recognizing the recurrent neoantigen nucleophosmine 1 (NPM1) presented on HLA-A*02:01 (ref. ^29,30^) and T cell clones targeting the fusion core-binding factor subunit β (CBFB)–myosin heavy chain 11 (MYH11) in the context of HLA-B*40:01 (ref. ^31^) showed some efficacy in patient-derived xenograft (PDX) mouse models with low AML engraftment. However, no neoantigen-specific TCR has yet been shown to efficiently target primary human AML in in vivo models in which high and increasing leukemic burden is observed.

**Fig. 1:**
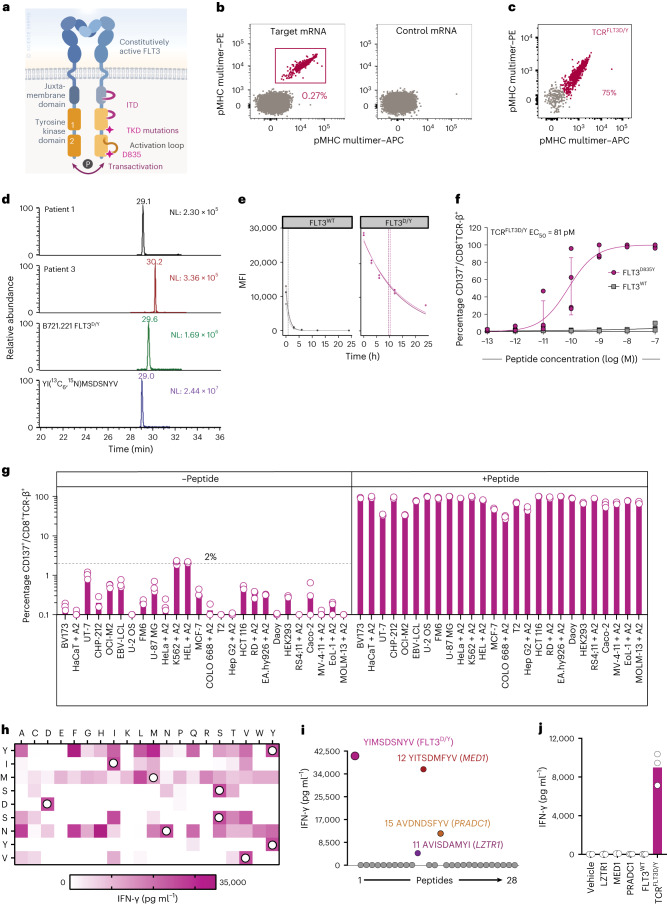
TCR^FLT3D/Y^ cells specifically recognize mutated peptide with high sensitivity in an HLA-A2-restricted manner and do not show off-target reactivity. **a**, Schematic illustration of FLT3. TKD, tyrosine kinase domain. **b**, Naive CD8^+^ T cells co-cultured with autologous HLA-A2^+^ mRNA-transfected moDCs stained with FLT3^D/Y^ pMHC multimers. **c**, CD8^+^ T cells transduced to express TCR^FLT3^^D/Y^ stained with FLT3^D/Y^ pMHC multimers (Gating strategy in Extended Data Fig. 3b). **d**, Parallel reaction-monitoring analysis, targeting the FLT3^D835Y^ peptide (*m*/*z* = 1,091.4389^1+^) in primary AML cells from two patient samples and the B721.221 cell line transduced to express FLT3^D835Y^ and HLA-A2. NL = normalization level. **e**, Off-rates for FLT3^WT^ or FLT3^D/Y^ peptide binding to HLA-A2 measured by flow cytometry. Vertical lines indicate calculated half-lives in each experiment. Dots represent mean fluorescence intensity (MFI) values of intact pMHC complexes on fluorescent particles at the indicated time points (h) (one replicate per experiment, *n* = 3 independent experiments). **f**, Activation of TCR^FLT3D/Y^ cells (CD137^+^) co-incubated with peptide-pulsed K562 cells. Data points are from *n* = 4 donors transduced to express TCR in *n* = 3 independent experiments, with each circle representing the mean of three technical replicates per donor, shown as mean ± s.e.m. **g**, Activation of CD8^+^ TCR^FLT3D/Y^ cells co-incubated with HLA-A2^+^ cell lines with or without FLT3^D/Y^ peptide. Results are from one experiment representative of *n* = 4 (BV173, CHP-212, EBV-LCL, K562, Daoy, RS4;11), *n* = 3 (HaCaT, U-2 OS, FM6, U-87 MG, HeLa, MV-4-11, EoL-1, MOLM-13) or, for the remaining cell lines, *n* = 2 independent experiments using different T cell donors; data points represent *n* = 3 technical replicates. The suffix + A2 denotes that cell lines were transduced with HLA-A*02:01, whereas remaining cell lines naturally express it. Connecting lines in **f** and bars in **g** show mean. The dashed line in **g** shows the highest level of activation by cell lines alone. **h**–**j**, IFN-γ produced by TCR^FLT3D/Y^ cells co-incubated with K562 cells loaded with peptides from the mimotope library (**h**) or pulsed with the peptides that were predicted as potentially cross-reactive from the in silico search (**i**) or transfected with mRNA constructs encoding 30–32-mer peptides with the candidate cross-reactive peptide inducing reactivity (shown in **i**) in the middle, flanked by its naturally occurring sequence, or transfected with mRNA encoding the FLT3^D/Y^ epitope or FLT3^WT^ (**j**). White circles in **h**, amino acids of the FLT3^D/Y^ peptide. Positive reaction for IFN-γ, 5,000–35,000 pg ml^−1^. LZTR1, leucine zipper-like post-translational regulator 1; MED1, mediator complex subunit 1; PRADC1, protease-associated domain-containing protein 1. Data in **h**–**j** are from one of *n* = 2 independent experiments, and individual data points represent one (**h**,**i**) or three (**j**) technical replicates. Source data

Recurrent driver mutations in *FLT3* occur in approximately one-third of patients with de novo AML as internal tandem duplications (ITD) in the juxtamembrane domain or point mutations in the activation loop of the tyrosine kinase domain (Fig. 1a), which are screened for in routine diagnostics of AML^32^. Although, in most cases, *FLT3* mutations are secondary events in leukemogenesis^33^, they are associated with accelerated clonal expansion and disease progression, and treatment with the tyrosine kinase inhibitor (TKI) midostaurin in patients with *FLT3* mutations receiving standard induction chemotherapy does significantly increase long-term survival^34^. This indicates that *FLT3*-mutated AML clones have a survival advantage during standard therapy, expanding to drive relapse unless effectively eradicated, but the efficacy of TKIs in eliminating *FLT3*-mutated clones remains variable^35–37^.

Because of varying length, ITDs do not encode shared neoantigens. Mutations in the D835 position of FLT3 represent the most frequent point mutations in *FLT3* (7–10% of patients with AML), with aspartic acid (D) to tyrosine (Y) being by far the most common amino acid substitution, although other substitutions can also occur^32^. The effect of TKIs on the D835Y mutation (FLT3^D835Y^) is limited by this mutation conferring primary and secondary resistance to first- and second-generation type II TKIs^35–37^.

We recently demonstrated that healthy donor T cells provide a rich source of neoantigen-reactive TCRs^38,39^. Here, we used this technology to identify a TCR reactive to a peptide from FLT3^D835Y^. The TCR (TCR^FLT3D/Y^) is restricted by HLA-A*02:01 (hereafter HLA-A2), expressed in approximately 50% of people of European, Middle Eastern or North African ancestry. We demonstrate that T cells redirected with TCR^FLT3D/Y^ (TCR^FLT3D/Y^ cells) specifically and efficiently eliminate primary human AML cells harboring FLT3^D835Y^ in vitro and in vivo, while sparing cells expressing wild-type (WT) FLT3.

## Results

### Inducing FLT3^D835Y^-reactive T cells from healthy donor T cells

**Extended Data Fig. 1:**
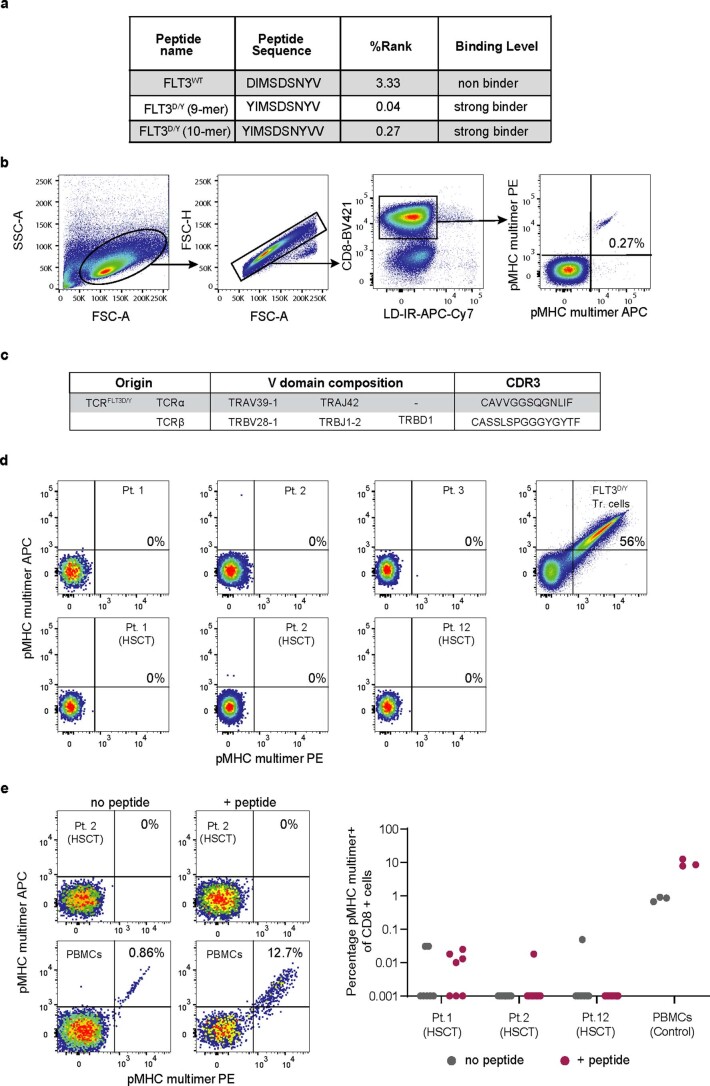
T cells reactive to FLT3^D/Y^ HLA-A2 can be induced by culture of naïve healthy donor T cells but are not identified among memory T cells from AML patients in diagnostic samples or following HSCT. **(a)** Predicted binding affinity of FLT3^D/Y^ and FLT3^WT^ peptides using the NetMHC-4.0 algorithm. Peptides with affinity <50 nM (EL%Rank <0.500) classify as strong binders, 50 nM<affinity<500 nM (EL%Rank <2.000) as weak binders and affinity >500 nM as non- binders^85^. **(b)** Gating strategy to identify CD8^+^ T cells staining as double positive events for pMHC multimers (APC- and PE-conjugated) complexed with the FLT3^D/Y^ peptide. The three left plots show gates used for FSC/SSC, singlets and Live/Dead Fixable Near- IR^neg^/CD8^+^ events. The plot to the right shows multimer positive events in co-culture of naïve healthy donor CD8^+^ T cells with autologous moDCs transfected with target mRNA. **(c)** The TCR^FLT3D/Y^ sequence. **(d)** Staining of samples from AML patients taken at point of diagnosis (top row, left three panels) and post allo-HSCT (bottom row) with pMHC multimers. TCR^FLT3D/Y^ transduced T cells were used as a positive control for multimer staining (top right). **(e)** Staining of samples obtained from patient 2 after allo-HSCT following 5 days of *in vitro* expansion in absence (left) and presence (right) of the FLT3^D/Y^ peptide (top panels). As positive control for *in vitro* expansion of memory T cells, TCR^FLT3D/Y^ T cells were spiked into autologous healthy PBMCs under the same conditions (bottom panels). Inset numbers represent the percentage of pMHC multimer^+^ cells out of CD8^+^ cells. The graph to the right shows data for all three patients and positive controls. Data shown are from one experiment with each dot representing a technical replicate (*n* = 7 for pt 1, *n* = 12 for pt 2, *n* = 8 for pt 12 and *n* = 3 for PBMC healthy donor). Source data

Naive T cells from a large number of HLA-A2-positive healthy blood donors (*n* = 16) were co-cultured with autologous monocyte-derived dendritic cells (moDCs) electroporated with mRNA encoding a 9-mer and a 10-mer peptide with the FLT3^D835Y^ mutation in position 1, predicted to be strong binders to HLA-A2 (Extended Data Fig. 1a). The sequence was flanked by 9–11 amino acids at the N-terminal and C-terminal end (target mRNA, encoding KICDFGLAR**Y**IMSDSNYVVRGNVRLARLP) or control mRNA. After 10 d of co-culture, cells were stained with dual-color peptide–MHC (pMHC) multimers complexed with the nonameric or decameric peptide in which the FLT3^D/Y^ mutation was in position 1. Multimer^+^CD8^+^T cells reactive with the 9-mer were detected in only one donor and only in the culture primed with the target mRNA and were sorted. Only one functional TCR sequence (TCR^FLT3D/Y^) was identified from two clones and from 55 sorted, sequenced single cells (Fig. 1b and Extended Data Fig. 1b,c).

By contrast, memory cytotoxic T lymphocytes reactive to FLT3^D835Y^ HLA-A2 were not identified among peripheral blood (PB) mononuclear cells (PBMCs) from diagnostic AML samples (*n* = 3) or from samples after allo-HSCT with HLA-matched donors (*n* = 3), directly after thawing or following a short 5-d in vitro culture in the presence of 100 nM of the FLT3^D/Y^ mutant peptide to expand memory T cells (Extended Data Fig. 1d,e).

### TCR^FLT3D/Y^ recognizes mutant peptide with high peptide–HLA stability

**Extended Data Fig. 2:**
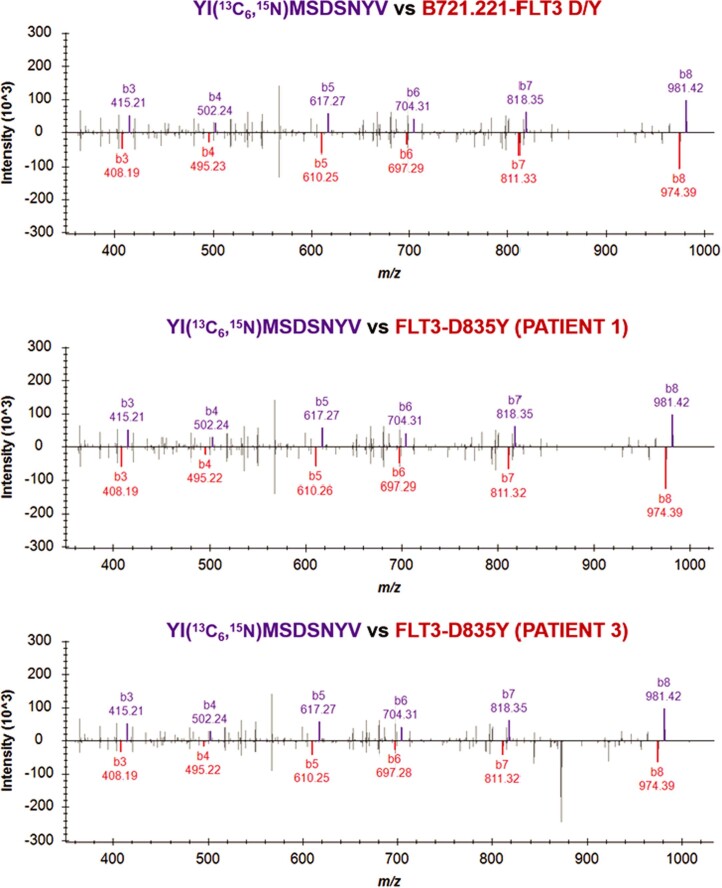
Tandem mass spectra of endogenous vs isotopically labelled peptide. Mirror image plots comparing the fragmentation spectra of endogenous FLT3^D/Y^ peptide to its isotopically labelled counterpart YI(13C6,15 N)MSDSNYV. Spectra obtained from immunoprecipitated HLA derived from the B721.221 cell line retrovirally transduced with a minigene encoding the D835Y mutation, and from patient 1 and patient 3, are displayed.

**Extended Data Fig. 3:**
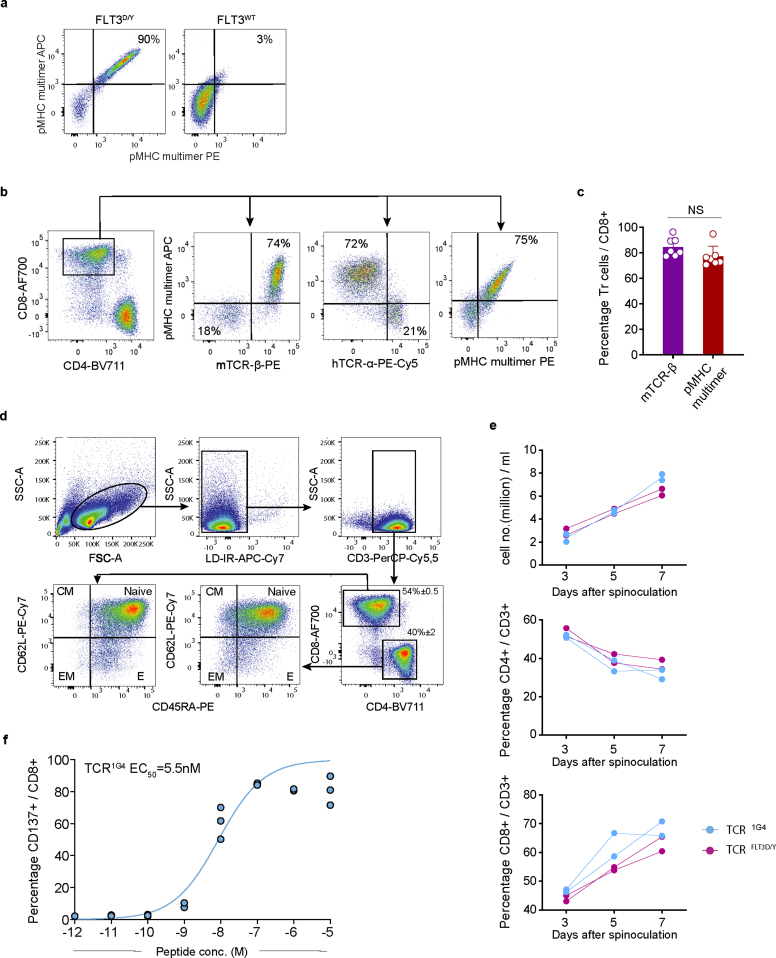
TCR^FLT3D/Y^ cells maintain a predominantly naïve phenotype following expansion. **(a)** Staining of TCR^FLT3D/Y^ cells with pMHC multimers complexed with either the FLT3^D/Y^ or the FLT3WT peptide (each multimer conjugated to both APC and PE, gating strategy shown in Extended Data Fig. 1b. Data shown is from one representative donor out of two stained in one experiment. **(b)** Gating strategy for TCR^FLT3D/Y^ cells. Panels show gating on: CD8^+^ cells labeled with the APC pMHC multimer and antibodies reactive to mouse TCR-β or human TCR-α (middle panels), and CD8^+^ cells staining positively for APC and PE-labeled pMHC multimers complexed with the FLT3^D/Y^ peptide (right plot). **(c)** Percentage of mTCR-β^+^ cells or pMHC multimer^+^ cells among CD8^+^ cells following transduction with the TCR^FLT3D/Y^. Each data point represents a different HLA-A2^pos^ donor (*n* = 7 donors from 3 independent experiments). Data are analyzed by unpaired, two-tailed Student’s t-test and *p* < 0.05 was considered statistically significant. **(d)** Gating strategy to identify differentiation stages of TCR-transduced T cells three days after spinoculation. Top panels show gating on FSC/SSC^hi^, Live/Dead Fixable Near-IR^neg^, CD3^+^ events. Bottom panels show gating on CD4^+^ or CD8^+^ populations and subsequent identification of naïve, central memory (CM), effector (E) and effector memory (EM) cells as defined by expression of CD45RA and CD62L analyzed in two donors in one experiment. **(e)** Expansion of HLA-A2^pos^ PB T cells from two donors transduced in parallel with the TCR^FLT3D/Y^ or TCR^1G4^ following indicated days after retroviral transduction. Data are from one experiment and dots represent one technical replicate for each HLA-A2^pos^ PB T cell donor. **(f)** Activation of TCR^1G4^ cells measured as upregulation of ^CD137^ after co-incubation with peptide-pulsed (NY-ESO-1 peptide SLLMWITQC) BV173 cells (K562 cells were not used, in contrast to Fig. 1f, as they express NY-ESO-1). EC_50_ = half maximal effective concentration (linear curve fitting). Data shown are from one experiment with one (out of two) T cell donors with individual data points representing technical replicates (*n* = 3). Source data

The TCR^FLT3D/Y^ sequence was efficiently expressed in third-party PB T cells by retroviral transduction (Fig. 1c). Binding to HLA-A2 as well as endogenous processing and presentation of the peptide was demonstrated by immunopeptidomics and targeted mass spectrometry analysis of primary AML cells from two patients, using a monoallelic cell line overexpressing the FLT3^D835Y^ mutation as a positive control (Fig. 1d, with ion chromatograms extracted using the precursor → b2, b3, b4, b5, b6, b7 and b8 fragment ion transactions, and Extended Data Fig. 2). We previously demonstrated that peptide–HLA stability is strongly predictive of neoantigen immunogenicity^38^, and the complex of HLA-A2 and the mutated peptide had an almost tenfold longer half-life than that of the WT peptide complexed with HLA-A2 (mean of 9.9 versus 0.83 h, *n* = 3) (Fig. 1e). In agreement with this, TCR^FLT3D/Y^ cells stained brightly with pMHC multimers presenting mutant, but not WT, peptide (Extended Data Fig. 3a). A similar fraction of TCR^FLT3D/Y^-redirected CD8^+^ T cells stained positively with FLT3^D/Y^ pMHC multimers and anti-mouse TCR-β–PE (reactive to the mouse constant region introduced into the TCR^40^) and negatively with anti-human TCR-α, indicating preferential pairing of the introduced TCR-α and TCR-β chains and suppression of the endogenous TCR (Extended Data Fig. 3b,c). Both CD8^+^ and CD4^+^ T cells exhibited a predominantly naive profile and expanded similarly to T cells transduced to express a control TCR in vitro, the clinically applied NY-ESO1-specific 1G4 (TCR^1G4^) (ref. ^41^), indicating lack of fratricide (Extended Data Fig. 3d,e).

### TCR^FLT3D/Y^ cells show high peptide sensitivity and specificity

TCR^FLT3D/Y^ cells recognized K562 target cells transduced to express HLA-A2 and pulsed with picomolar concentrations of the mutant peptide (half-maximal effective concentration (EC_50_) = 81 pM) and displayed no reactivity against the corresponding WT peptide (Fig. 1f). By comparison, TCR^1G4^ cells recognized the cognate NY-ESO1 peptide with an EC_50_ of 5.5 nM (Extended Data Fig. 3f), in agreement with previous data showing an EC_50_ of 7.7 nM^42^. Furthermore, TCR^FLT3D/Y^ cells showed no or negligible reactivity to a panel of 26 HLA-A2^+^ cell lines of different tissue origins, unless preloaded with mutant peptide, suggesting a high degree of peptide and HLA specificity (Fig. 1g).

**Extended Data Fig. 4:**
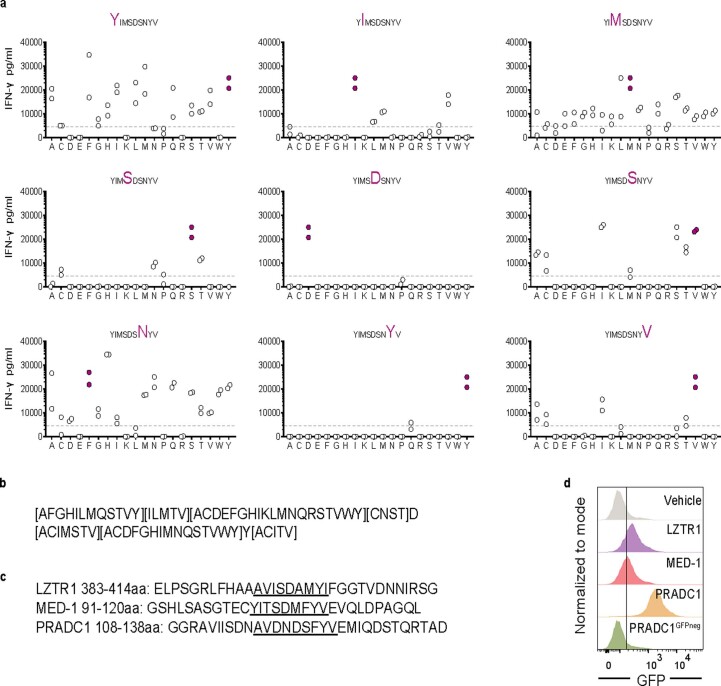
Mapping of peptide specificity does not reveal unintended targets to which the TCR^FLT3D/Y^ cells cross-react. **(a)** Graphs depicting IFN-γ response of TCR^FLT3D/Y^ cells to K562 cells loaded with individual peptides from mimotope library containing a total of 161 nine-mers for the FLT3^D/Y^ peptide, at a concentration of 10^−8^ M. Purple dots in each graph represent response to the FLT3^D/Y^ peptide. Substituted amino acid in the original peptide is highlighted. IFN-γ concentration range for positive reactions was 5000–35000 pg/mL (cut-off indicated by horizontal lines). Graphs show results from two independent experiments that were performed, one technical replicate (dot) per peptide and experiment. **(b)** Peptide reactivity motifs for FLT3^D/Y^ that were queried in the ScanProsite search tool against human proteome databases. Amino acids in square brackets [] indicate alternatives that are allowed for the given position in the peptide motifs. **(c)** mRNA-encoded amino acid sequences(30-32mers) derived from the sequence of the human proteins LZTR1, MED1 and PRADC1, with the candidate cross-reactive peptide identified in the ScanProsite database underlined. **(d)** Expression of mRNA encoding LZTR1, MED1 and PRADC1 sequences following electroporation of HLA-A2^pos^ K562 cells as measured by GFP-reporter fluorescence using flow cytometry. Source data

We next mapped the fine specificity of TCR^FLT3D/Y^. TCR^FLT3D/Y^ cells were screened for interferon (IFN)-γ production in response to target cells loaded with each peptide in a library of 161 peptides representing single-amino acid-substituted variants of the cognate peptide (Fig. 1h and Extended Data Fig. 4a). Peptide motifs harboring any ‘permitted’ alternative amino acids in each position were queried in the human proteome databases UniProtKB/Swiss-Prot by employing the ScanProsite tool (https://prosite.expasy.org/scanprosite/) (Extended Data Fig. 4b), identifying 28 additional 9-mers in the human proteome potentially recognized by TCR^FLT3D/Y^ cells (Supplementary Table 1). However, only three of these peptides (peptide 11, AVISDAMYI, derived from *LZTR1*; peptide 12, YITSDMFYV, derived from *MED1*; and peptide 15, AVDNDSFYV, derived from *PRADC1*) activated TCR^FLT3D/Y^ cells (Fig. 1i). The three genes encoding proteins harboring the potentially cross-reactive peptide sequences are ubiquitously expressed according to the HPA database (https://www.proteinatlas.org/). A lack of reactivity observed in response to the panel of 26 HLA-A2^+^ cell lines (Fig. 1g) therefore suggested that the peptides are not processed and presented. To further exclude this, we generated mRNA constructs encoding 30-mer peptides with the cross-reactive peptide in the middle flanked by the natural respective protein sequences and a green fluorescent protein (GFP) reporter (Extended Data Fig. 4c). Co-cultures demonstrated that TCR^FLT3D/Y^ cells did not react to K562 HLA-A2^+^ target cells electroporated with the mRNA constructs encoding peptides derived from *LZTR1*, *MED1* or *PRADC1* (Fig. 1j and Extended Data Fig. 4d), indicating that these peptides are not naturally processed and presented on HLA-A2. In agreement with this, none of the 28 candidate peptides were found in the HLA ligand database^43^.

### TCR^FLT3D/Y^ cells kill primary FLT3^D835Y^ AML cells in vitro

**Extended Data Fig. 5:**
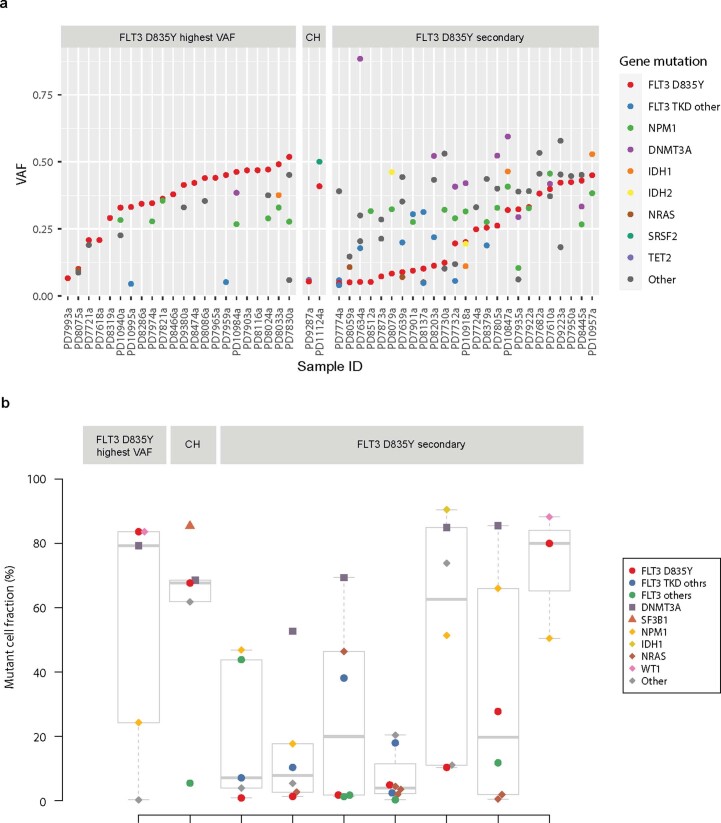
Re-analysis of published mutation data for 58 FLT3 D835Y positive AML patient samples shows high VAF for FLT3 D835Y in a large fraction of patients. **(a)** Mutation data (SNVs and indels) from AML patients reported in Papaemmanuil *et al*, 2016 NEJM were downloaded from https://www.cbioportal.org/. VAF was estimated from reported alternative allele reads divided by sequencing depth for the position. Patients harboring a FLT3 D835Y mutation were selected for in-depth analysis and displayed here. Patients are sorted from right to left within each subsection in descending order of FLT3 D835Y VAF. The following genes were considered as initiating events in clonal hematopoiesis (CH): *DNMT3A, TET2, ASXL1, PPM1D, JAK2, SF3B1, SRSF2, TP53*, *GNA*S and *GNB1*. **(b)** Mutation data from FLT3 D835Y positive AML patients (*n* = 9) reported in Morita *et al*, 2020 Nat Com were downloaded. The fraction of cells with mutations (mutant cell fraction) in each AML patient with mutations are plotted in indicated colors. Patients were categorized into 3 groups; FLT3 D835Y largest, only preceded by mutations in *DNMT3A, TET2*, and/or *ASXL1* (DTA) and/or splicing factor mutations (*SF3B1, SRAF2*, and *U2AF1*) which are closely related with clonal hematopoiesis ‘CH’, and the rest of cases with FLT3 D835Y mutation according to the cell fraction of mutations. Interquartile range (IQR) and median values are shown. The dashed lines indicate 1.5xIQR and the dots indicate outliers.

Reanalysis of publicly available DNA sequencing data of 49 patients with AML and the FLT3^D835Y^ mutation^15^ demonstrated that the FLT3^D835Y^-mutated clone is frequently dominant (Extended Data Fig. 5a). The FLT3^D835Y^ mutation had the highest variant allele frequency (VAF) among identified recurrent driver single-nucleotide variants (SNVs) and indels in 22 patients (45%). In two additional patients, it was only preceded by a recurrent *SRSF2* and *TET2* mutation, known to occur in normal individuals with clonal hematopoiesis (CH) and requiring additional driver mutations to transform to AML^44^. Similarly, reanalysis of single-cell DNA-sequenced AMLs showed in two of nine patients with the FLT3^D835Y^ mutation that it had the highest VAF or was secondary only to CH-associated mutations^45^ (Extended Data Fig. 5b). In sum, this analysis of 58 FLT3^D835Y^-mutated patients with AML indicates that FLT3^D835^ is frequently clonal and that it might constitute an AML-initiating or transforming mutation.

**Fig. 2:**
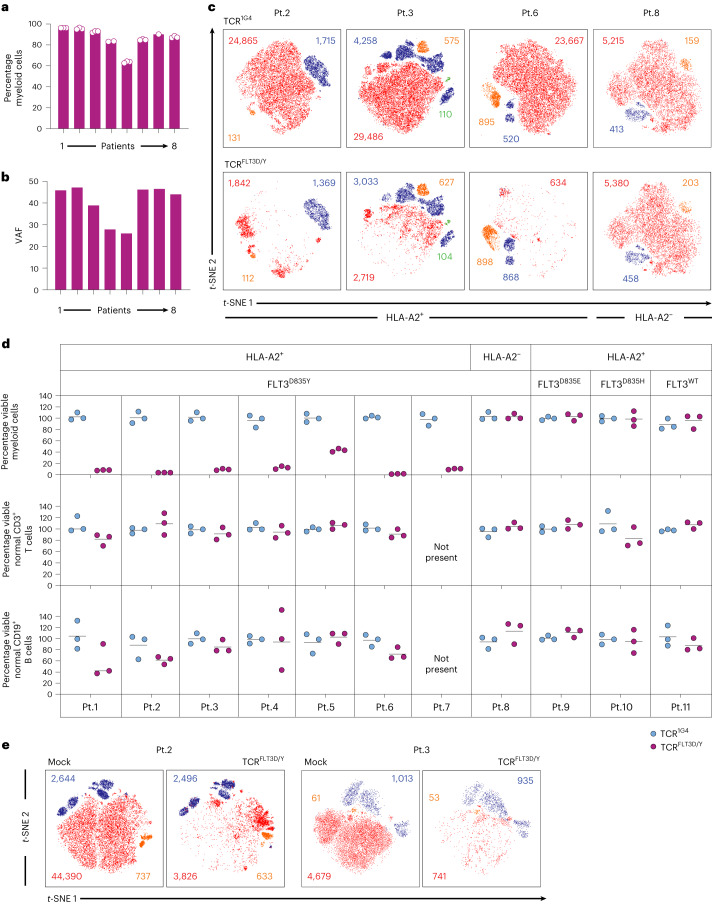
TCR^FLT3D/Y^ cells efficiently kill primary AML cells harboring the FLT3^D835Y^ mutation in vitro but spare normal lymphoid cells. **a**, Percentage myeloid cells of live leukocytes for patients (Pt.) 1–8 with AML; gating strategy is shown in Extended Data Fig. 6a. Dots represents technical replicates from one representative experiment as described in **d**. **b**, PB or BM FLT3^D/Y^ VAF for patients 1–8 as determined by next-generation sequencing. **c**, Representative *t*-distributed stochastic neighbor embedding (*t*-SNE) plots showing live primary myeloid cells (CD3^−^CD19^−^CD20^−^ events) in red, T cells (CD3^+^) in blue, B cells (CD19^+^CD20^+^) in orange and normal CD34^+^lin^−^ progenitor cells in green from *n* = 3 representative HLA-A2^+^ FLT3^D/Y^ patients (patients 2, 3 and 6) with AML and one HLA-A2^−^ FLT3^D/Y^ patient (patient 8) following 72 h of co-culture with TCR^1G4^ (negative control, top) or TCR^FLT3D/Y^ cells (E:T ratio, 1:2) as quantified by flow cytometry. Cells transduced to express TCR were excluded from analysis as CellTrace Violet (CTV)-positive events. **d**, Diagnostic samples from 11 patients with AML and the FLT3^D/Y^ (patients 1–8), FLT3^D/E^ (patient 9) or FLT3^D/H^ (patient 10) mutation or FLT3^WT^ (patient 11) (all HLA-A2^+^ except patient 8), analyzed as described in **c**. Each dot represents the fraction of live myeloid cells, B cells or T cells after co-culture with TCR^FLT3D/Y^ cells (purple) in percent mean of the corresponding numbers in cultures treated with TCR^1G4^ cells (blue). Data points represent *n* = 3 technical replicates, and horizontal lines show means. Data shown are from one experiment representative of two to four experiments performed for each patient sample (*n* = 1 only for patient 7). **e**, *t*-SNE plots of PB diagnostic samples from patients 2 and 3 with AML showing live myeloid, T and B cells (color coded as in Fig. 2c) after 72 h of co-culture with autologous T cells transduced to express TCR^FLT3D/Y^ or the mock control. Inset numbers in **c**,**e** denote absolute event counts of the indicated cell populations. The gating strategy is shown in Extended Data Fig. 6e. Source data

**Extended Data Fig. 6:**
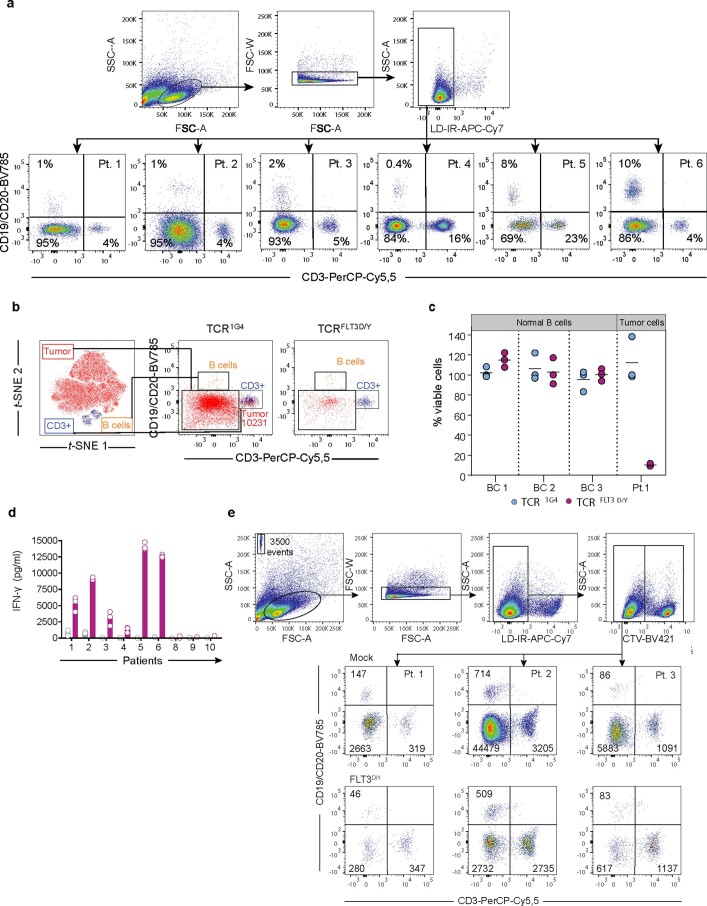
TCR^FLT3D/Y^ cells are activated by, and efficiently kill, primary AML cells expressing FLT3^D/Y^. **(a)** Flow cytometry plots showing gating strategy to identify cell subsets in AML patient samples from PB (patient 2-6) or BM (patient 1). Cells are gated on FSC/SSC^hi^, single, Live/Dead Fixable Near-IR events (top row), showing the fractions of CD3^+^ T cells, CD19^+^CD20^+^ B cells and myeloid cells, defined as CD3^−^CD19^−^CD20^−^ events in the bottom row. **(b)** Populations were overlaid on a *t*- SNE plot (patient 1) with designated colors as indicated. Inset numbers show event counts for myeloid cells after co-culture with TCR^1G4^ or TCR^FLT3D/Y^ cells, quantified as shown in e. **(c)** Quantification of normal CD19^+^ B cells isolated from *n* = 3 healthy blood donors (Buffy coat (BC) 1 - BC 3) and tumor cells from patient 1 (positive control) after performing the flow cytometry-based cytotoxicity assay for 72 h. Data points represent *n* = 3 technical replicates from one experiment and horizontal lines show mean. **(d)** Bar graph showing IFN-γ response of Mock (gray) and TCR^FLT3D/Y^ cells (purple) after 24 h co-culture with HLA-A2^pos^ patient cells expressing the FLT3 D835Y (Pt.1-6), FLT3 D835E (Pt.9) or FLT3 D835H (Pt. 10), or Pt.8 cells expressing FLT3 D835Y but being HLA-A2^neg^. Data points represent *n* = 3 technical replicates and bars show mean. Data shown are from one experiment representative of at least two performed for each patient sample. **(e)** Gating strategy for flow cytometry cytotoxicity assay to quantify viable cells in subpopulations from AML patients 1-3 after 72 h of co-culture with autologous T cells either expressing TCR^FLT3D/Y^ (bottom row) or mock-transduced. Transduced T cells were labeled with cell-trace violet (shown in upper right plot) prior to co-culture with AML cells to distinguish them from T cells in the AML samples. Patient cell subsets are gated on FSC/SSC, singlets, Live/Dead Fixable Near-IR^neg^, CTV^neg^ events. Numbers indicate absolute counts for CD3^+^, CD19^+^/CD20^+^ and myeloid cells after co-culture, as determined by addition of fluorescent beads (10,000) into each well and where 3,500 beads were acquired for flow cytometry analysis (shown in upper left plot). Source data

We next explored the specificity and efficacy with which TCR^FLT3D/Y^ cells killed AML cells from 11 patients (Supplementary Table 2). DNA sequencing of mononuclear samples dominated by myeloid cells (Fig. 2a and Extended Data Fig. 6a) showed high FLT3^D835Y^ clonal involvement in the eight FLT3^D835Y+^ patients (Fig. 2b and Supplementary Table 3). TCR^FLT3D/Y^ cells killed myeloid cells from patients 1–7 with AML effectively at effector:target (E:T) ratios as low as 1:2 (mean, 87%; range, 53.3–98.7%; Fig. 2c,d). CD3^+^ T cells and CD19^+^CD20^+^ B cells were not significantly affected (Fig. 2c,d and Extended Data Fig. 6b), as expected in view of low clonal involvement^46^. However, B cell counts were very low (Extended Data Fig. 6a,b), and we therefore also investigated the effect of TCR^FLT3D/Y^ cells on isolated B cells from healthy donors, which demonstrated a lack of killing (Extended Data Fig. 6c). The HLA restriction of TCR^FLT3D/Y^ was confirmed by lack of killing of myeloid HLA-A2^−^ FLT3^D835Y^ patient cells (patient 8; Fig. 2c,d). Specificity for the D835Y substitution of FLT3 was demonstrated by lack of recognition of AML samples with alternative amino acid substitutions in the D835 position (patients 9 and 10) or expressing FLT3^WT^ (patient 11) (Fig. 2d and Supplementary Table 2). Robust and specific IFN-γ production was observed upon co-incubation of TCR^FLT3D/Y^ cells with HLA-A2^+^ FLT3^D835Y^ patient cells (patients 1–6; Extended Data Fig. 6d). Finally, TCR^FLT3D/Y^ cells derived from patients with AML killed autologous leukemia cells with similar efficacy and selectivity as third-party T cells, mimicking the clinical setting (Fig. 2e and Extended Data Fig. 6e). In most cases, two to four experiments were performed per patient sample, with only one experiment performed due to a limited amount of material in a few instances, as described in the figure legends.

### TCR^FLT3D/Y^ cells efficiently target human leukemia cells in vivo

**Extended Data Fig. 7:**
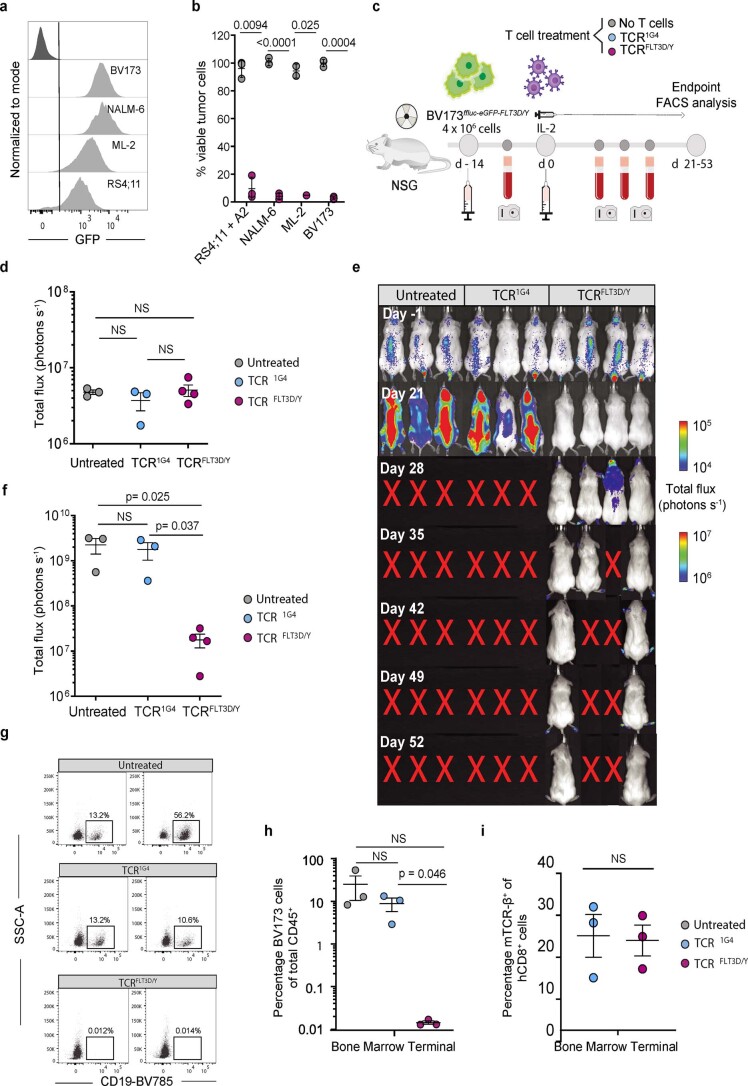
TCR^FLT3D/Y^ cells efficiently target leukemia in a xenograft mouse model. **(a)** Flow cytometry histograms showing expression of FLT3^D/Y^ as measured by GFP- reporter fluorescence in transduced AML and B-ALL cell lines. Negative control (non-transduced BV173 cells) in top histogram. **(b)** Remaining viable FLT3^D/Y^-transduced, HLA-A2+ cells (purple dots) after 24 h co-culture with TCR^FLT3D/Y^ cells (E:T ratio of 1:2), in percent of corresponding numbers following treatment with mock-transduced T cells (grey dots), quantified by flow cytometry. +A2 denotes that HLA-A2 was introduced by transduction. Data points are from *n* = 3 independent experiments with each dot representing the mean of *n* = 3 technical replicates in each experiment and are shown as mean ± s.e.m. Gating strategy and quantification as shown in Extended Data Fig. 6e. **(c)** Schematic overview of the BV173^D835Y^ *in vivo* model. **(d)** Bioluminescence imaging (BLI) analysis of NSG mice day 13 after BV173^D835Y^ cell injection, one day prior to T-cell therapy. Data shown are from one experiment, with mice grouped into untreated (*n* = 3 mice), TCR^1G4^ (*n* = 3 mice) and TCR^FLT3D/Y^ (*n* = 4 mice) cell treated. **(e)** BLI of BV173^D835Y^ engrafted leukemic cells in mice on indicated days relative to treatment with TCR^1G4^ or TCR^FLT3D/Y^ cells, or no treatment. **(f)** Quantification of BV173^D835Y^ engrafted leukemic cells in mice, 21 days after treatment with TCR^1G4^ or TCR^FLT3D/Y^ cells or left untreated. **(g)** Flow cytometry plots showing BM tumor burden in two untreated and two TCR^1G4^ cell-treated mice at time of sacrifice (d 21), as well as two TCR^FLT3D/Y^ cell treated mice sacrificed at end of experiment (d 53). **(h)** Percentage of bone marrow BV173^D835Y^ leukemic cells in mice treated with TCR^1G4^ or TCR^FLT3D/Y^ cells, or left untreated, out of total mouse and human CD45^+^ cells (21-53 days after treatment start). **(i)** Percentage of TCR-β^+^ cells out of human CD8^+^ cells in the BM of mice analyzed at point of sacrifice. Data in **d, f, h, i** are presented as mean ± s.e.m, are from one experiment with each dot representing an individual mouse, and are analyzed by unpaired, two-tailed Student’s t-test. *P* values are shown and *p* < 0.05 was considered statistically significant. Source data

We initially investigated whether TCR^FLT3D/Y^ cells recognized endogenously presented antigen in a small experiment using an in vivo xenograft mouse model engrafted with a leukemia cell line. Because FLT3^D835Y^ leukemic cell lines are not commercially available, we introduced the mutation into different leukemia cell lines and demonstrated that TCR^FLT3D/Y^ cells killed >95% of leukemic cells in 24 h at low E:T ratios (1:2) (Extended Data Fig. 7a,b). BV173 FLT3^D835Y^-expressing cells (cell line origin, B cell precursor leukemia) were next transplanted into NOD.Cg-*Prkdc*^scid^ *Il2rg*^*tm1Wjl*^/SzJ (NSG) mice (Extended Data Fig. 7c,d). All mice in the control groups (untreated (*n* = 3) and TCR^1G4^ cells (*n* = 3)) were killed after 21 d due to high leukemic burden, at which time leukemic cells were undetectable in the TCR^FLT3D/Y^ cell-treated group (*n* = 4) (Extended Data Fig. 7e,f). Among four mice receiving the therapeutic TCR, two survived for the duration of the experiment (53 d) and one remained free of leukemia but was killed on day 41 due to graft-versus-host disease, whereas one relapsed on day 28 and was found dead on day 32 (Extended Data Fig. 7e). Upon termination, no leukemic cells were detected in the bone marrow (BM) of the two surviving TCR^FLT3D/Y^ cell-treated mice (Extended Data Fig. 7g,h), while transduced T cells in the BM persisted (Extended Data Fig. 7i).

### TCR^FLT3D/Y^ cells efficiently eliminate primary AML in PDX models

To investigate the in vivo efficacy of TCR^FLT3D/Y^ cells in disease-relevant models, we next treated mice engrafted with primary AML cells in different PDX models.

#### Model 1

**Fig. 3:**
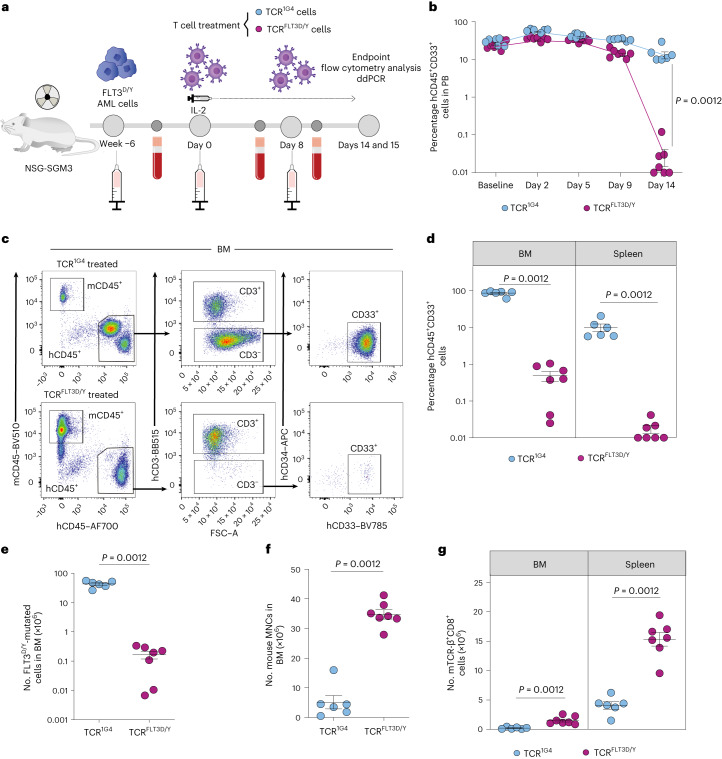
TCR^FLT3D/Y^ cells efficiently target primary AML in mice with high leukemic burden. **a**, Schematic overview of the PDX in vivo model with FLT3^D835Y^-mutated primary AML cells from patient 7. **b**, Percentage of human hCD45^+^CD33^+^ cells in PB at baseline (1 d before T cell infusion) and on the indicated days after infusion with TCR^1G4^ (*n* = 6 mice) or TCR^FLT3D/Y^ (*n* = 7 mice) cells. Numbers were adjusted for hCD3^+^ T cells. **c**, Representative flow cytometry plots of viable single BM mononuclear cells (MNCs) from TCR^1G4^ (top) and TCR^FLT3D/Y^ (bottom) cell-treated NSG-SGM3 mice stably engrafted with primary AML FLT3^D/Y^ cells from patient 7. **d**, Percentage of hCD45^+^CD33^+^ cells in the BM and spleen at terminal analysis 15 d after T cell infusion. Numbers were adjusted for hCD3^+^ T cells. **e**, Number of FLT3^D835Y^-mutated BM hCD45^+^CD3^−^ cells determined by ddPCR. **f**,**g**, Number of mouse (m)CD45^+^ cells in BM (**f**) and mTCR-β^+^CD8^+^ cells in the BM and spleen (**g**) at the endpoint. All data are presented as mean ± s.e.m. and were generated from one experiment including six mice treated with TCR^1G4^ cells and seven mice treated with TCR^FLT3D/Y^ cells. Each dot represents one mouse, and statistical analysis was performed with two-tailed Mann–Whitney test. *P* values are shown, and *P* < 0.05 was considered statistically significant. Source data

**Extended Data Fig. 8:**
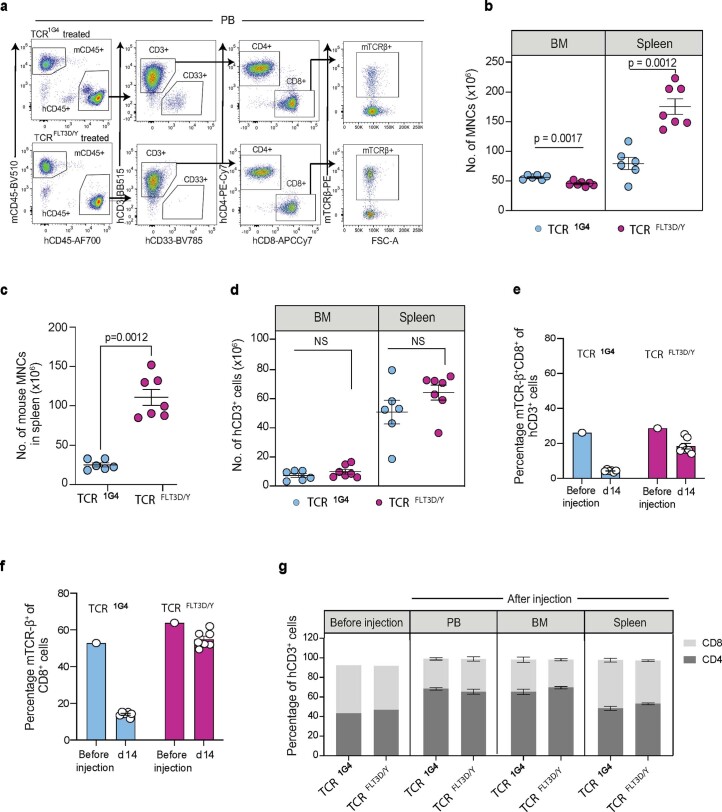
TCR^FLT3D/Y^ cells persist *in vivo* and mediate recovery of mouse hematopoiesis in patient 7 PDX model. **(a)** Representative FACS plots of viable single PB MNCs from TCR^1G4^ (top) and TCR^FLT3D/Y^ (bottom) T cell-treated NSG-SGM3 mice stably engrafted with primary AML FLT3 D835Y cells from patient 7 at 14 days post T cell infusion. Equivalent gating as shown was also used for spleen. **(b)** Number of total MNCs in BM and spleen of TCR T cell treated mice. **(c)** Number of mouse MNCs (mCD45+ cells) in spleen. **(d)** Number of total hCD3+ cells in BM and spleen. **(e-f)** Percentage of mTCR-β^+^CD8^+^ cells of hCD3^+^ cells (e) and percentage of mTCR-β^+^ cells of hCD8+ cells (f) in T cell samples used for injection and at indicated days after T cell injection, in PB**. (g)** Distribution (%) of CD4 and CD8 cells within the human CD3^+^ T cell samples used for injection, and in PB, BM and spleen 14-15 days after injection into mice. All data are from terminal analysis 15 days post T-cell infusion if not otherwise stated. The data are presented as mean ± s.e.m. of *n* = 6 individual mice treated with TCR^1G4^ cells and of *n* = 7 individual mice treated with TCR^FLT3D/Y^ cells, measured in one experiment. Each dot represents one mouse and statistical analysis was performed with two-tailed Mann-Whitney test. *P* values are shown and *p* < 0.05 was considered statistically significant. Source data

NSG-SGM3 mice highly engrafted with primary FLT3^D835Y^ AML cells from patient 7 (24.5% ± 2.2% human CD33^+^ cells in PB) (Fig. 3a,b and Supplementary Tables 2 and 3) were treated with TCR^FLT3D/Y^ (*n* = 7) or control TCR^1G4^ (*n* = 6) cells. Serial analysis of PB demonstrated a maintained high CD33^+^ engraftment in TCR^1G4^-treated mice, whereas CD33^+^ cells were virtually eliminated by day 14 in all TCR^FLT3D/Y^-treated mice (Fig. 3b and Extended Data Fig. 8a). Mice were closely monitored for potential allo-reactivity mediated by endogenous TCRs of the T cells transduced to express TCR derived from a third-party donor, which could otherwise confound the anti-tumor reactivity mediated by TCR^FLT3D/Y^. Thus, terminal analysis of all TCR^1G4^-treated and TCR^FLT3D/Y^-treated mice was performed on day 15 after T cell infusion when tumor burden started to decline slightly also in control mice (Fig. 3b). BM analysis demonstrated high CD33^+^ AML engraftment in TCR^1G4^-treated mice (mean, 86.9% ± 5.3%), which was reduced to a mean of 0.5% ± 0.2% in TCR^FLT3D/Y^-treated mice, with a similar reduction in the spleen (Fig. 3c,d and Supplementary Table 4). An almost complete elimination of FLT3^D/Y^ AML cells in the BM was confirmed by droplet digital PCR (ddPCR) analysis, quantifying clonally involved cells (Fig. 3e and Supplementary Table 5). Importantly, efficient targeting of human AML cells in TCR^FLT3D/Y^-treated mice resulted in recovery of mouse hematopoiesis, which was severely suppressed by high leukemic burden in TCR^1G4^-treated mice (Fig. 3f and Extended Data Fig. 8b,c). T cells transduced to express TCR were detected in the BM, spleen and PB of all mice throughout the experiment (Fig. 3g and Extended Data Fig. 8d–g).

#### Model 2

**Fig. 4:**
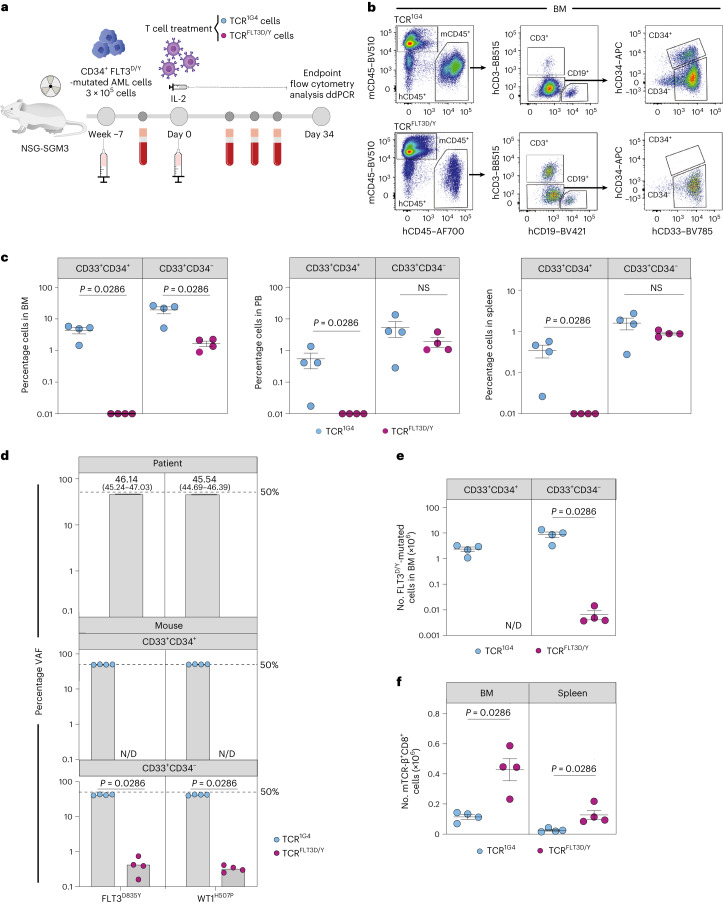
TCR^FLT3D/Y^ cells eliminate primary CD34^+^ AML in vivo. **a**, Schematic overview of the PDX in vivo model with FLT3^D835Y^-mutated primary AML cells from patient 1. **b**, Representative flow cytometry plots of BM from TCR^1G4^ (top) and TCR^FLT3D/Y^ (bottom) cell-treated NSG-SGM3 mice stably engrafted with primary AML FLT3^D/Y^ cells from patient 1. Equivalent gating was also used for PB and the spleen. **c**, Percentage of hCD45^+^CD33^+^CD34^+^ and hCD45^+^CD33^+^CD34^−^ cells in the BM, PB and spleen at the endpoint (day 34 after T cell infusion) of TCR^1G4^ (*n* = 4 mice) or TCR^FLT3D/Y^ (*n* = 4 mice) cell-treated mice. Numbers were adjusted for hCD3^+^ T cells. NS, not significant. **d**, Percentage VAF determined by ddPCR of FLT3^D835Y^ and WT1^H507P^ driver mutations in primary BM cells from patient 1 (top) and hCD45^+^CD33^+^CD34^+^ (middle) and hCD45^+^CD33^+^CD34^−^ (bottom) cells from TCR cell-treated mice. N/D, not analyzed due to insufficient hCD45^+^CD33^+^CD34^+^ cells. Numbers show VAF and 95% confidence intervals. The dashed line at 50% indicates 100% clonality unless loss of heterozygosity. No significant differences in VAFs of the FLT3^D835Y^ and WT1^H507P^ mutations were observed. **e**, Number of FLT3^D835Y^-mutated hCD45^+^CD33^+^CD34^+^ and hCD45^+^CD33^+^CD34^−^ cells in the BM as determined by ddPCR. N/D, not detected due to lack of hCD45^+^CD33^+^CD34^+^ cells. **f**, Number of mTCR-β^+^CD8^+^ cells in the BM and spleen at the endpoint. All data are presented as mean ± s.e.m. from terminal analysis 34 d after T cell infusion from one experiment including four mice treated with TCR^1G4^ cells and another four mice treated with TCR^FLT3D/Y^ cells. Each dot represents one mouse, and statistical analysis was performed with two-tailed Mann–Whitney test. *P* values are shown, and *P* < 0.05 was considered statistically significant. Source data

**Extended Data Fig. 9:**
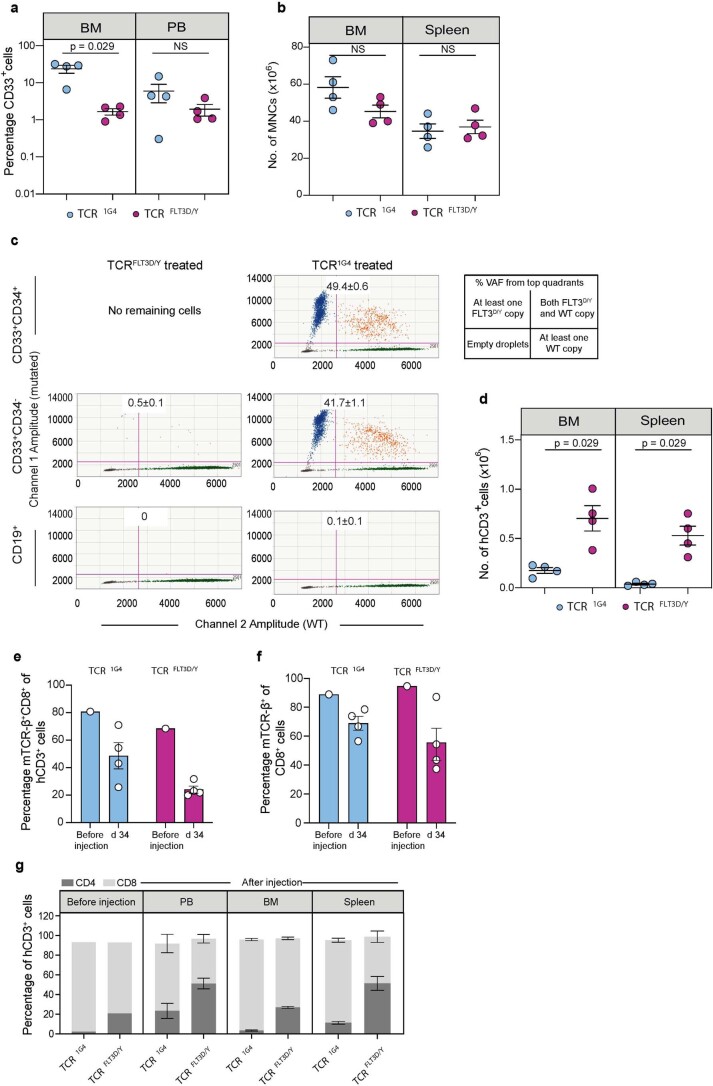
TCR^FLT3D/Y^ cells eliminate FLT3^D835Y^ leukemia cells and persist *in vivo* in patient 1 PDX model. **(a)** Percentage of CD33^+^ cells in BM and PB at endpoint (day 34 post T cell infusion) of TCR^1G4^ cell (*n* = 4) or TCR^FLT3D/Y^ cell (*n* = 4) treated mice. Numbers adjusted for hCD3^+^ T cells. **(b)** Number of total MNCs in BM and spleen of TCR T cell treated mice. **(c)** Representative ddPCR plots of BM from TCR cell-treated mice. Numbers in quadrants represent %VAF (mean ± s.e.m.) of the FLT3^D/Y^ in BM among hCD45^+^CD33^+^CD34^+^, hCD45^+^CD33^+^CD34^−^ and hCD45^+^CD19^+^ cells from TCR^FLT3D/Y^ cell-treated (left) and TCR^1G4^ cell-treated (right) mice. ‘No remaining cells’ due to elimination of hCD45^+^CD33^+^CD34^+^ cells in TCR^FLT3D/Y^ cell-treated mice. **(d)** Number of total hCD3^+^ cells in BM and spleen. **(e-g)** Percentage of mTCR-β^+^CD8^+^ cells of hCD3^+^ cells (e) and percentage of mTCR-β^+^ cells of hCD8^+^ cells (f) in sample used for injection and at indicated days after T cell infusion in PB. **(g)** Distribution (%) of CD4 and CD8 cells within the human CD3^+^ T cell samples analyzed prior to injection, and in PB, BM and spleen of individual mice at endpoint. All data are from terminal analysis 34 days post T-cell infusion unless otherwise stated. The data are presented as mean ± s.e.m. of *n* = 4 individual mice treated with TCR^1G4^ cells and of *n* = 4 mice treated with TCR^FLT3D/Y^ cells, measured in one experiment. Each dot represents one mouse and statistical analysis was performed with two-tailed Mann-Whitney test. *P* values are shown and *p* < 0.05 was considered statistically significant. Source data

Primary xenografts from a second FLT3^D835Y^-mutated AML sample engrafted in NSG-SGM3 mice (patient 1, Supplementary Table 2) were investigated for response to TCR^FLT3D/Y^ cells (Fig. 4a). Unlike patient 7 (Fig. 3c), this AML co-expressed CD34, a stem and progenitor marker characteristic of CD34^+^ AMLs^47^, for which CD34^+^ cells in contrast to CD34^−^ cells have been shown to possess leukemia-propagating activity^48,49^. Control TCR-treated mice had relatively low levels of PB human CD33^+^ engraftment 34 d after T cell injection (mean, 6.0% ± 3.1% human (h)CD45^+^CD33^+^ cells), whereas BM engraftment was much higher (mean, 23.7% ± 5.8%) (Fig. 4b, Extended Data Fig. 9a,b and Supplementary Table 6). In TCR^FLT3D/Y^-treated mice, this was reduced to 1.9% ± 0.7% in PB and 1.7% ± 0.3% in the BM (Extended Data Fig. 9a,b). Terminal BM analysis on day 34 after T cell injection revealed distinct populations of CD33^+^CD34^−^ (mean, 19.4% ± 4.8%) and CD33^+^CD34^+^ cells (mean, 4.4% ± 1.0%) in control TCR^1G4^-treated mice (Fig. 4b,c). In TCR^FLT3D/Y^-treated mice, some CD33^+^CD34^−^ cells persisted in the BM although at greatly reduced levels (mean 1.7% ± 0.3%), whereas CD33^+^CD34^+^ cells were completely eliminated. Similar findings were observed in PB and the spleen (Fig. 4c and Supplementary Table 6).

DNA sequencing confirmed that cells engrafted in NSG-SGM3 mice were represented by the same exonic mutations as those detected in AML blasts from the BM of patient 1, with FLT3^D835Y^ and WT1^H507P^ representing the only detected recurrent driver mutations (Supplementary Table 7). Further quantification by ddPCR demonstrated that FLT3^D835Y^ and WT1^H507P^ mutations represented the dominating clone, with a VAF > 45% in patient 1 as well as in engrafted NSG-SGM3 mice (Fig. 4d). Notably, among the remaining CD33^+^CD34^−^ BM cells of TCR^FLT3D/Y^-treated mice, the fraction of FLT3^D835Y^-mutated cells was reduced to only 0.9% ± 0.3% as compared with 83.4% ± 2.1% in TCR^1G4^-treated mice. This translates into more than a 1,300-fold reduction in CD33^+^CD34^−^ FLT3^D/Y^ AML cells in TCR^FLT3D/Y^-treated mice (Fig. 4d,e, Extended Data Fig. 9c and Supplementary Table 8). Human T cells including T cells transduced to express TCR were detected in the BM, spleen and PB of all treated mice throughout the course of the experiment (Fig. 4f and Extended Data Fig. 9d–g). Cells from patient 1 also reconstituted CD19^+^ B lymphocytes in engrafted mice (Fig. 4b), and, in agreement with previous studies^46^, these were not part of the FLT3^D/Y^ leukemic clone (Extended Data Fig. 9c and Supplementary Table 8).

#### Model 3

**Fig. 5:**
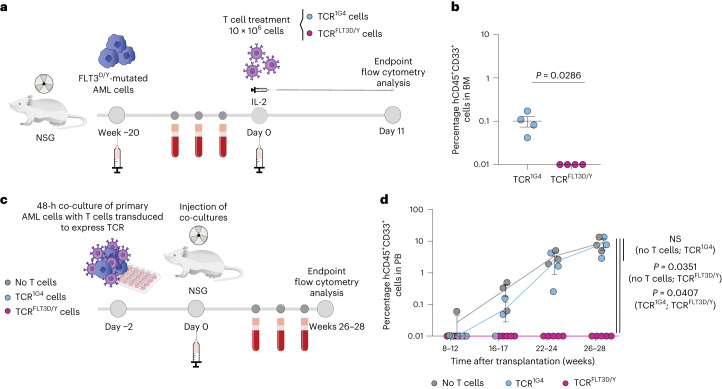
TCR^FLT3D/Y^ cells efficiently kill primary AML in an MRD setting and eliminate leukemia-propagating cells. **a**, Schematic overview of the MRD PDX in vivo model with FLT3^D835Y^-mutated primary AML cells from patient 1. **b**, Percentage of hCD45^+^CD33^+^ cells in the BM of NSG mice engrafted with low levels of AML after treatment with TCR^1G4^ (*n* = 4 mice) or TCR^FLT3D/Y^ (*n* = 4 mice) cells 11 d after T cell infusion. Numbers were adjusted for hCD3^+^ T cells. Data are presented as mean ± s.e.m. and were generated from one experiment. Each dot represents one mouse, and statistical analysis was performed with two-tailed Mann–Whitney test. **c**, Schematic overview of the PDX in vivo model with FLT3^D835Y^-mutated primary AML cells from patient 1 after in vitro targeting with TCR^1G4^ or TCR^FLT3D/Y^ cells. **d**, Percentage of hCD45^+^CD33^+^ cells in PB at the indicated time after transplantation of primary AML cells from patient 1 following 48 h of co-culture without T cells (*n* = 3 mice) or with TCR^1G4^ (*n* = 3 mice) or TCR^FLT3D/Y^ (*n* = 5 mice) cells. Data are presented as mean ± s.e.m. and were generated from two independent experiments. Each dot represents one mouse, and statistical analysis was performed by multilevel linear regression using the R package ‘lmerTest’ (further described in the Methods). *P* values are shown, and *P* < 0.05 was considered statistically significant. Source data

To establish a PDX model mimicking minimal residual disease (MRD)^50^, secondary transplantations of mice engrafted with AML cells from patient 1 were performed into NSG mice, a mouse strain that, in contrast to NSG-SGM3 mice, does not specifically enhance human myeloid lineages. In accordance with results from the NSG-SGM3 model, we observed efficient elimination of FLT3^D/Y^ primary AML cells also in this MRD setting (Fig. 5a,b and Supplementary Table 9).

#### Model 4

**Extended Data Fig. 10:**
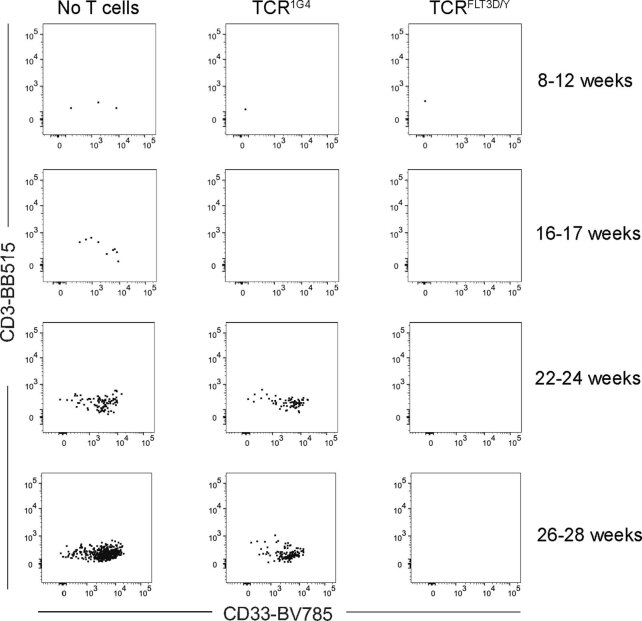
Lack of *in vivo* T-cell expansion upon transplantation of primary AML cells co-cultured with TCR^1G4^ or TCR^FLT3D/Y^ T cells. Representative FACS profiles of engrafted human CD45^+^ cells in the PB of mice analyzed at indicated weeks after transplantation of all cells remaining after 48 hours *in vitro* culture of AML cells without T cells (left), AML cells co-cultured with TCR^1G4^ cells (middle) or AML cells co-cultured with TCR^FLT3D/Y^ cells (right). Profiles are from one mouse per treatment group from one experiment out of two performed.

TCR^FLT3D/Y^ treatment resulted in undetectable levels of CD34^+^ FLT3^D/Y^ AML cells in the PB, spleen and BM of all mice engrafted with AML cells from patient 1, compatible with complete elimination of FLT3^D/Y^ AML-propagating cells in vivo through specific TCR^FLT3D/Y^ targeting. More definitive support for this would, however, require longer follow-up of mice to allow for potential outgrowth of rare and therapy-resistant AML stem cells that might have escaped T cell recognition. A long follow-up time was not possible in models 1–3 due to the continued presence of TCR T cells having endogenous TCR repertoires that cause allo-reactivity and xenoreactivity over time. Moreover, to establish whether all AML-propagating cells have been eliminated by TCR^FLT3D/Y^ cells requires assessment in the absence of TCR^FLT3D/Y^ T cells. To circumvent these limitations, AML cells from patient 1 were cultured with TCR^FLT3D/Y^ or TCR^1G4^ cells or without T cells in vitro for 48 h before transplantation into NSG mice (Fig. 5c). The very few human T cells to which the AML cells were exposed in vitro did not persist in vivo (Extended Data Fig. 10). The mice did not receive interleukin (IL)-2 infusions. Potential AML development could therefore be followed for 28 weeks. During this time, mice injected with AML cells from control cultures without T cells or with control TCR^1G4^ cells showed progressive and high leukemic engraftment. By contrast, no detectable engraftment was observed at any time in any mice transplanted with AML cells co-cultured with TCR^FLT3D/Y^ cells (Fig. 5d and Extended Data Fig. 10). Collectively, these experiments demonstrate that TCR^FLT3D/Y^ cells can efficiently target and eliminate FLT3^D835Y^-mutated in vivo AML-propagating cells.

## Discussion

In this study, we identify a TCR recognizing an epitope from the recurrent driver mutation D835Y in the tyrosine kinase domain of FLT3 in AML, presented on the prevalent HLA-A2 allele. This TCR, selected from healthy donor T cell repertoires, mediated highly specific and efficient killing of primary AML cells harboring the FLT3^D835Y^ mutation in vitro and in vivo. Different clinically relevant aspects of therapeutic effects were demonstrated in different in vivo mouse PDX models. This included an almost complete removal of mutated CD34^−^ AML in model 1 with high engraftment and complete removal of CD34^+^ AML in model 2 with lower levels of reconstitution. In a third PDX model, TCR^FLT3D/Y^ cells rejected patient AML cells in a setting resembling MRD. TCR^FLT3D/Y^ cells thus efficiently eliminated leukemia with both high and low disease burden. AML cells that are CD34^+^ have previously been shown to propagate leukemia in vivo^49^. Here, we demonstrated, in a fourth PDX model followed for 7 months after AML injection, that TCR^FLT3D/Y^ cells in vitro can specifically and efficiently target and kill leukemia-propagating CD34^+^ FLT3^D835Y^-mutated AML cells. Studies in humans would, however, be required to confirm that TCR^FLT3D/Y^ cells could achieve this also in vivo. In sum, this suggests that a therapeutic TCR targeting a single, shared neoantigen has the potential of eliminating the reservoir of leukemia stem cells in AML.

The prospect of treating large patient groups with cytotoxic T cells expressing a single TCR targeting public neoantigens expressed by prevalent HLA molecules has emerged as an attractive therapeutic possibility^51^. However, although a large number of recurrent hotspot mutations are known, it has proven difficult to identify HLA-bound peptides from tumor samples by immunopeptidomics^52,53^. One reason might be that driver mutations that are poorly presented to the immune system provide a survival advantage. Another possibility is that mass spectrometry has insufficient sensitivity for detection of many neoantigenic peptides. We were, however, able to identify the FLT3^D/Y^ neoepitope in two AML patient samples by targeted mass spectrometry, starting with a large number of leukemia cells. T cells are, on the other hand, the most sensitive tools available for detection of pMHC complexes^54^. Our technology using mRNA-transduced dendritic cells to prime naive T cells from healthy donors provides the means to identify neoepitopes and reactive TCRs in a single assay^38,39^. Presentation of the FLT3^D/Y^ neoepitope on leukemic cells was confirmed by efficient killing of primary HLA-A2^+^ AML cells expressing FLT3^D835Y^, but not the less frequent FLT3^D835E^ or FLT3^D835H^ mutations or FLT3^WT^, by TCR^FLT3D/Y^ cells, further demonstrating TCR specificity.

To date, most efforts to isolate neoantigen-specific TCRs employ strategies in which memory T cell responses in patient material are interrogated^11,55,56^. Identification of TCRs with sufficient affinity is essential for clinical impact^41,57^. A hurdle might be induction of T cell tolerance due to long-standing co-evolution between cancer and the immune system in the absence of inflammation and lack of sufficient priming. This might contribute to the low spontaneous reactivity that tumor-infiltrating T cells show to the large repertoire of predicted neoantigens in melanoma^11^. In support of this, immune responses to neoantigen vaccines seem to be dominated by de novo responses induced in naive T cells^58–60^. The possibility of inducing neoantigen-specific responses from naive patient-derived T cells is, however, limited by the amount of blood that can be collected and by T cell repertoires that might be reduced by foregoing therapy. In an earlier study, we showed that healthy donor-derived T cells recognized fivefold more neoantigens than tumor-infiltrating lymphocytes of patients with melanoma^38^. Here, we used the same approach to identify TCR^FLT3D/Y^, which recognizes peptide at picomolar concentrations and mediates efficient cytotoxicity against primary AML cells in vitro and in vivo. High antigen sensitivity, indicated by EC_50_ values below 10 nM, appears to be an important factor for TCRs in effectively killing cancer cells, as previously demonstrated by us^27^ and others^9,41,61,62^ Similarly, Foy et al., in their publication in *Nature*, discussed the potential association between low EC_50_ values observed for personalized neoantigen-reactive TCRs in their study and the limited clinical efficacy observed^12^.

We primed T cells from 16 healthy donors to identify T cells recognizing the YIMSDSNYV peptide presented by HLA-A2. This indicates low immunogenicity of the peptide, consistent with lack of recognition by the investigated AML patient T cells, although a limited number of cells were screened for reactivity. In sum, this demonstrates the advantage of accessing large, healthy donor T cell repertoires. We previously demonstrated that peptide–HLA stability is an important predictor of neoantigen immunogenicity^38^. Here, we showed that the off-rate for the FLT3^835–843^ peptide bound to HLA-A2 was significantly slower when substituting the WT amino acid D in position 1 with a Y. This is consistent with other studies showing that increased stability of the neoantigen relative to the corresponding WT peptide leads to sustained antigen presentation and increased T cell recognition^63,64^. Testing for potential off-target reactivity is essential to detect possible safety concerns before clinical use^65^. Mapping of TCR fine specificity using a library of single-amino acid-substituted peptides followed by a bioinformatic screen did not identify cross-recognized peptides, although this does not completely exclude potential recognition of peptide sequences that are unrelated to the cognate peptide. TCR^FLT3D/Y^ cells did not, however, react to a panel of 26 HLA-A2^+^ cell lines of different tissue origins and spared normal blood cells and AML cells expressing the FLT3^WT^ sequence or alternative FLT3^D835^ mutations.

Treatment with TCR^FLT3D/Y^ cells would be limited to the 3–4% of patients that, in addition to expressing the mutation also express HLA-A2 in the European-descended population^32^. While recurrent *FLT3* mutations are frequently known to represent secondary and accelerating AML mutations^15,33,66^, our reanalysis of 58 published FLT3^D835Y^-mutated AML patient samples^15,45^ demonstrated that this FLT3 mutation frequently is clonal and, in some cases, also might be the initiating mutation or secondary only to a CH mutation. These data are in agreement with the high VAF for FLT3^D835Y^ in all included patients with AML harboring this mutation, where the only selection criterion upfront was the presence of the mutation. In sum, this highlights the therapeutic and potentially curative potential of eradicating FLT3^D835Y^-mutated AML clones. Furthermore, in cases in which TKIs effectively target FLT3 ITD but select for AML subclones with FLT3 point mutations, including FLT3^D835Y^, resistant to first-generation as well as second-generation TKIs^35–37^, FLT3^D835Y^-mutation-specific TCR treatment could be combined with TKIs. As part of a paradigm in which combination regimens are tailored based on tumor molecular profiles, TCR therapies targeting specific recurrent point mutations provide a potential means for highly efficacious eradication of specific tumor clones.

TCRs can access recurrent mutations currently inaccessible to CARs. Moreover, TCRs might have inherent advantages relative to CARs that improve T cell persistence and antigen sensitivity^67^. Today, TCR-based therapies are mostly applied in the form of genetically modified T cells, although soluble bispecific TCR engagers have emerged as new opportunities for off-the-shelf therapies at lower cost^68^. The results presented here show that a TCR targeting a single shared neoantigen generated from a healthy donor can provide highly efficacious and specific cancer treatment in vivo in multiple disease-relevant models, paving the way for future off-the-shelf, tumor-specific immunotherapies.

## Methods

This study was approved by the Regional Committee for Medical and Health Research Ethics South-East Norway (2018/879, 2018/1246 and 2015/2357), the Institutional Review Board and the Data Protection Officer, Oslo University Hospital, the Swedish Ethical Review Authority, Stockholm (EPN 2017/2085-31/2) and the Ethical Committee in Central Denmark Region (1-45-70-88-21) and was performed in accordance with the Declaration of Helsinki. The Norwegian Food Safety Authority (application ID 17500) and Stockholms Djurförsöksetiska nämnd (17978-2018) approved all animal experiments.

### Primary patient cells, healthy blood donor cells and cell lines

Buffy coats (PBMCs) from healthy donors were provided by the blood bank of Oslo University Hospital, and PB or BM MNCs from patients with leukemia were isolated from cryopreserved, biobanked material (ethical approvals 2018/879 and 2018/1246). MNCs derived by density-gradient centrifugation (Axis-Shield) were stained to determine HLA-A2 expression by flow cytometry. To confirm the presence of FLT3^D835Y^, genomic DNA was extracted (QIAGEN DNeasy purification kit) from patient primary cells, and samples were sequenced using the TruSight Myeloid panel (Illumina). Information regarding the patients’ sex (reported in Supplementary Table 2) was not considered during the design of the study but was part of the overall clinical information obtained at the hospital. No sex- or gender-based analysis was performed because each patient is shown individually in the study.

Epstein–Barr virus-transformed lymphoblastoid cell lines (EBV-LCL) were generated from HLA-A2^+^ and HLA-A2^−^ PBMCs as described previously^27^. All other cell lines were gifted or obtained from the American Type Culture Collection (ATCC) or the German Collection of Microorganisms and Cell Cultures (DSMZ) as indicated in the Nature Portfolio Reporting Summary. Authentication was performed by short tandem repeat DNA profiling by Labcorp DNA Identification Lab (formerly Genetica, https://celllineauthentication.com/). Cell line cultures were grown in humidified cell incubators containing 5% CO_2_ at 37 °C using media according to provider guidelines and were tested frequently for potential mycoplasma contamination.

### Minigene design

For generation of T cell responses, a minigene was designed to encode predicted epitopes (https://services.healthtech.dtu.dk/service.php?NetMHC-4.0) containing the FLT3^D835Y^ mutation, codon optimized and synthesized by GenScript. Subsequently, it was cloned into the pCIpA102 vector for in vitro mRNA transcription using the RiboMAX Large Scale RNA production system (Promega), as previously described^69,70^. The minigene encoded the FLT3 amino acid sequence KICDFGLAR**Y**IMSDSNYVVRGNVRLARLP (FLT3^D835Y^ 9-mer and 10-mer are underlined and the mutation in position 1 is shown in bold).

### Induction of antigen-specific T cells

Monocytes from HLA-A2^+^ healthy donors were isolated on day −4 using CD14-reactive microbeads and the autoMACS Pro Separator (Miltenyi Biotec). The CD14^−^ PBMC fraction was cryopreserved for later use. The monocytes were then cultured for 3 d in CellGro GMP DC medium (CellGenix) with 1% (vol/vol) human serum (HS; Trina biotech), 1% (vol/vol) penicillin–streptomycin (P/S; Sigma-Aldrich), 50 IU ml^−1^ IL-4 (PeproTech) and 800 IU ml^−1^ GM-CSF (Genzyme). Subsequently, moDCs were matured for 14–16 h by adding lipopolysaccharide (Sigma-Aldrich) and IFN-γ (PeproTech) to final concentrations of 10 ng ml^−1^ and 100 IU ml^−1^, respectively. On day −1, naive CD8^+^ T cells were isolated from the autologous CD14^−^ cryopreserved PBMCs by use of the autoMACS Pro Separator and a CD8^+^ T cell-isolation kit, into which CD45RO- and CD57-reactive beads (Miltenyi Biotec) were added. On day 0, moDCs were collected, electroporated with mRNA and co-cultured with naive T cells in DC–T cell medium with 30 ng ml^−1^ IL-21 (PeproTech) at a DC:T cell ratio of 1:4. After 10 d, co-cultures were screened for the presence of FLT3^D/Y^ pMHC multimer-reactive CD8^+^ T cells. pMHC multimers labeled with PE and APC were prepared in house as described previously^71,72^. Viable CD8^+^pMHC^+^ T cells (double positive for PE- and APC-conjugated pMHC multimers) were sorted by flow cytometry.

### Expansion of memory T cells reactive to FLT3^D/Y^ in samples from patients with AML following HSCT

For in vitro expansion of potential memory T cells reactive to the FLT3^D/Y^-mutant peptide in patients with AML that had undergone HSCT, cryopreserved PB samples were thawed and resuspended in Iscove’s Modified Dulbecco’s Medium (IMDM) with 20% (vol/vol) FCS (Trina biotech) and 0.1 mg ml^−1^ DNase. Viable cells were resuspended at a concentration of 1 M ml^−1^ and pulsed with the FLT3^D/Y^ peptide at 100 ng ml^−1^ for 2 h at 37 °C. Cells were washed and resuspended at 3.75 million cells per ml in IMDM with 5% HS, 1× P/S and 20 U ml^−1^ IL-2 before culturing for 5 d. Identification of T cells reactive to the peptide was performed by staining with pMHC multimers as described above.

### Sorting and cloning of pMHC multimer^+^CD8^+^ T cells

PBMCs from three healthy donors were mixed at an equal ratio (1:1:1) and irradiated with 35 Gy, washed and resuspended in X-VIVO 20 medium (Lonza, BioNordika) with 5% HS and 1% P/S (T cell medium). A total of 0.2 × 10^6^ irradiated cells (feeders) were placed into tissue culture-treated 96-well plates and were supplemented with 100 μl T cell medium containing 2 μg ml^−1^ phytohemagglutinin (Remel Thermo Scientific), 80 ng ml^−1^ IL-2 (R&D Systems) and 4 ng ml^−1^ IL-15 (PeproTech). FLT3^D/Y^ co-cultures were then collected and stained with LIVE/DEAD Fixable Near-IR, anti-CD3 antibody, anti-CD8a antibody and PE- and APC-conjugated pMHC multimers, and, using the FACSAria II (BD Biosciences) cell sorter, CD8^+^, pMHC-double-positive multimer populations were sorted as single cells into 96-well plates containing feeders. After 7 d, cultures were supplied with fresh T cell medium containing 1,750 U ml^−1^ IL-2 and 4 ng ml^−1^ IL-15, and expanding clones were identified by microscopy. On day 14, growing clones were restimulated with feeder cells prepared as described above and stained with FLT3^D/Y^ pMHC multimers. To assess functionality after expansion, clones were stimulated with K562 cells pulsed with FLT3^D/Y^ or FLT3^WT^ peptides and assessed for CD137 upregulation.

### TCR sequencing

The sequences for paired TCR-α and TCR-β chains from two clones and 55 single cells reactive to FLT3^D/Y^ pMHC multimers were amplified as previously described but modified and adapted for the targeted amplification of transcripts encoding TCR-α and TCR-β (refs. ^27,38,55^). The MiXCR script was used to analyze sequencing data, and an in-house Python script TCR primer was used to reconstruct full-length TCR chains as described previously^55,73^. The output was manually verified using IMGT/V-QUEST^74^. For identified TCRs, codon-optimized sequences for TCR-α and TCR-β variable fragments were synthesized and cloned by GenScript.

### Gene transfer to human PBMCs and cell lines

HLA-A2^+^ healthy donor-derived and patient-derived PBMCs were transduced to express FLT3^D/Y^- and NY-ESO-1 (1G4)-specific TCRs, as detailed in ref. ^27^. Briefly, 2 × 10^6^ PBMCs per ml in CellGro GMP DC medium with 5% (vol/vol) HS, IL-7 and IL-15 (5 ng ml^−1^ each, PeproTech) were added to antibody-coated plates (anti-CD3 clone OKT3, eBioscience and anti-CD28 clone CD28.6, eBioscience) and incubated at 37 °C with 5% CO_2_ for 72 h. Retroviral supernatants were generated as described previously^27^. PBMCs were collected, resuspended in CellGro GMP DC medium with 5% HS, IL-7 and IL-15, mixed with retroviral supernatant, placed in non-tissue culture-treated six-well plates precoated with RetroNectin (20 μg ml^−1^, Takara) and spinoculated at 900*g* for 60 min twice on consecutive days. Transduction efficiency was determined after 3 d by staining with anti-mouse TCR-β chain antibody and/or the pMHC multimer followed by flow cytometry. Before functional experiments, cells were cultured for 48–72 h in CellGro GMP DC medium containing low concentrations of cytokines (0.5 ng ml^−1^ IL-7 and IL-15). Alternatively, cells were frozen for later experiments.

BV173, ML-2, RS4;11 and NALM-6 cell lines were transduced as described above using retroviral supernatant containing the FLT3^D/Y^ minigene. For in vivo experiments, the BV173 cell line was stably transduced to express the FLT3^D/Y^ minigene, firefly luciferase and GFP (hereafter, BV173^D835Y^). Complementary DNA encoding FLT3^D/Y^ and HLA-A2 was cloned into the pCIpA102 vector for production of mRNA, as previously described^70,71^

### Immunoprecipitation-targeted mass spectrometry analysis of FLT3 peptides presented on HLA

Monoallelic B721.221 cells expressing HLA-A2 were transduced to express the mutant FLT3 amino acid sequence VLVTHGKVVKICDFGLAR**Y**IMSDSNYVVRGNARLPVK. One hundred million cells from the B721.221 line and 400 million cells from patient samples (patients 1 and 3) were lysed in PBS containing 1% lauryl maltoside, 0.5 mM EDTA, 1 mM PMSF and Sigma protease inhibitors (1:200) for 1 h at 4 °C (1 ml lysis buffer per 100 million cells). The clarified cell lysates were then added to 200 µl of AminoLink Plus bead slurry (Thermo Fisher Scientific) coated with pan-HLA class I-specific antibody (W6/32, BioXCell) to enrich for HLA peptides^27^. The HLA-bound peptides were then sequentially eluted three times, each with 1 ml of 1% TFA. Peptide elutions were pooled and desalted using the Discovery DSC-C18 SPE column. The peptides were vacuum concentrated and dissolved in 25 μl of 3% acetonitrile containing 0.1% TFA, following spike-in with 200 pg of heavy isotope-labeled peptide (YI(^13^C_6_,^15^N)MSDSNYV). The peptide solution (5 μl) was analyzed using an EASY-nLC 1000 system (Thermo Fisher Scientific) connected to a Q Exactive HF mass spectrometer (Thermo Electron) equipped with a nano-electrospray ion source. For liquid chromatography separation, an EASY-Spray ES902 column (C18, 2-µm beads, 100 Å, 75-μm inner diameter) capillary with a bed length of 25 cm was used. A flow rate of 300 nl min^−1^ was employed with a solvent gradient of 7–35% B in 55 min to 90% B in 3 min. Solvent A was 0.1% formic acid, and solvent B was 0.1% formic acid–90% acetonitrile. The mass spectrometer was operated in parallel reaction-monitoring mode to specifically target the presence of endogenous FLT3 mutant (*m*/*z* = 1,091.4738^1+^) and spiked-in isotope-labeled peptide (*m*/*z* = 1,098.4928^1+^), eluting within a retention time window of 27–31 min, as determined using a synthetic analog. The MS/MS spectra using higher-energy collision-induced dissociation were acquired with a resolution of *R* = 15,000 after accumulation to a target of 1 × 10^5^. The normalized collision energy was set to NCE 27, and the isolation window was *m*/*z* = 2.0. The maximum allowed ion accumulation for the MS/MS spectrum was 120 ms. Raw data were analyzed using Xcalibur software and Skyline (MacCoss Lab Software).

### pMHC-stability assay

HLA-A2 molecules were prepared in house, as previously described^71,75,76^. The pMHC-stability assay was performed as previously described^38^ with minor modifications. UV-mediated peptide-exchange reactions were performed for 1 h, followed by incubation of the resulting product at 4 °C. The next day, streptavidin-coated beads were washed twice with PBS–1% Tween. The peptide–HLA monomers were coupled with the washed beads for 10 min at room temperature. After coupling, the beads were washed twice with PBS–1% Tween and resuspended in 200 μl. An aliquot of 20 μl beads was set aside for the 0-h time point, while the remaining beads were incubated at 37 °C. Twenty microliters of beads were collected at 3, 6, 12 and 24 h of incubation. After collecting at each time point, the beads were stained with 30 μl of anti-HLA-A2–PE antibody (343305, BioLegend, 1:100 dilution) for 10 min at room temperature. Samples from all time points were analyzed immediately after staining on a BD LSR II Flow Cytometer.

### Antibodies, dyes and flow cytometry

For surface antibody staining of human PB and BM cells, antibodies were added to cells for 15–20 min at 4 °C, followed by washing steps. For intracellular staining, cells were suspended in Cytofix/Cytoperm (BD Biosciences) solution for 20 min, washed with Perm/Wash buffer (BD Biosciences) and then stained with antibodies. For mouse PB, BM and spleen, cells were processed into a single-cell suspension as previously described^77^ and Fc receptor blocked (human, Miltenyi Biotec; mouse, produced by mouse hybridoma cell line clone 2.4 G2, ATCC, HB-197) for 10 min at 4 °C before staining with antibodies for 15–20 min at 4 °C. All fluorescently conjugated antibodies are described in Supplementary Table 10 and the Nature Portfolio Reporting Summary. In PDX mice, the percentage of myeloid cells was determined as a fraction of combined mouse and human leukocytes (mCD45^+^hCD45^+^), subtracting hCD3^+^ events accounting for infused T cells. Flow cytometry analysis was performed on the BD LSR II flow cytometer or the BD LSRFortessa machine (both BD Biosciences), while cell sorting was performed on the FACSAria Fusion cell sorter (BD Biosciences). Data were analyzed using FlowJo (TreeStar) or FACSDiva (BD Biosciences) software. To visually display flow cytometry data, we used an unsupervised nonlinear dimensionality-reduction algorithm such as *t*-SNE by using FlowJo (TreeStar) software.

### T cell-activation assays

T cells transduced to express TCR were co-cultured with cell lines or primary patient tumor cells at an E:T cell ratio of 1:2 (100,000:200,000 cells per well), and reactivity was investigated by measuring CD137 upregulation or IFN-γ release. When indicated, target cells were pulsed with FLT3^D/Y^ or FLT3^WT^ peptide (purities >90%) or 161 single-amino acid-substituted variants of the FLT3^D/Y^ peptide (purity >70%) (GenScript Biotech) for 1–2 h or electroporated with mRNA encoding either FLT3^WT^ or the FLT3^D/Y^ minigene. Cells were placed in round- or flat-bottom 96-well plates and, after 18–20 h of co-incubation, were centrifuged at 700*g* for 2 min. Supernatants were collected for measurement of IFN-γ levels by ELISA, while cells were washed and stained with anti-CD137 antibody. In some experiments, transduced T cells were labeled with 0.75 μM CTV to distinguish them from target cells. Reagents for the IFN-γ ELISA were acquired from BD Pharmingen or R&D Systems: mouse anti-human IFN-γ capture antibody (NIB42), Biotin Mouse Anti-Human IFN-γ-detection antibody (4S.B3), streptavidin–HRP, stabilized tetramethylbenzidine and hydrogen peroxide as substrate solutions, sulfuric acid as the stop solution and recombinant human IFN-γ protein as the standard. The assay was performed according to the manufacturer’s instructions.

### Flow cytometry-based cytotoxicity assay using cell lines as targets

Transduced cell lines stably expressing the FLT3^D/Y^ minigene were co-cultured with CTV-labeled T cells transduced to express TCR at an E:T cell ratio of 1:2 (75,000:150,000 cells per well) for 48 h in round-bottom 96-well plates in triplicates. Following co-culture, cells were collected, washed and stained with human anti-CD3, anti-CD8 and anti-CD4 antibodies and LIVE/DEAD NIR for 15–20 min. Cells were then washed and resuspended in FACS buffer containing 10,000 CountBright Absolute Counting Beads (Thermo Fisher). An equal number of bead events (3,500) were recorded from every well. Normalized data were reported as percentage of the mean of the number of viable tumor cells acquired from three parallel wells co-cultured with TCR^1G4^ or TCR^FLT3D/Y^ cells from each donor.

### Flow cytometry-based assays for T cell activation and cytotoxicity using primary human samples

PB or BM samples from patients were thawed and resuspended in IMDM with 20% (vol/vol) FCS (Trina biotech) and 0.1 mg ml^−1^ DNase. Cells were centrifuged at 200*g* for 15 min at room temperature and transferred to round-bottom 96-well plates for assays measuring CD137 upregulation on T cells transduced to express TCR or cytotoxicity on target cells. Normal CD19^+^ B cells were isolated from healthy donor buffy coat MNCs using CD19-reactive microbeads and the autoMACS Pro Separator (Miltenyi Biotec) and transferred to round-bottom 96-well plates for assays measuring cytotoxicity on target cells. Individualized antibody panels and gating strategies to identify malignant blasts and normal leukocyte populations were designed after reviewing diagnostic phenotyping available in the hospital records. Allogeneic or, for patients 1–3, also autologous patient-derived T cells transduced to express TCRs, were used in the experiments. Cells transduced to express TCR were prelabeled with CTV dye to distinguish them from target cells. For cytotoxicity assays, 75,000 T cells per well were co-incubated with 150,000 target cells in three parallel wells per condition for 72 h and then stained with individualized antibody panels for flow cytometry. CountBright Absolute Counting Beads were used as described above. Examples of the gating strategy used to identify live tumor cells in patients are shown in Extended Data Fig. 6a. For patients 1–6 and 8, myeloid cells were identified as CD3^−^CD19^−^CD20^−^, normal T cells as CD3^+^ and normal B cells as CD19^+^CD20^+^. Cells from patient 7 were obtained from Jackson Laboratory (stock ID J000106565), and the patient-specific phenotypic markers CD33 and CD19 were used to identify leukemia cells based on the characterization profile from the provider.

### TCR^FLT3D/Y^ cell activity in the xenograft leukemia cell line model

This study was approved by the Norwegian Food Safety Authority (application ID 17500) and performed in compliance with institutional guidelines and the 2010/63/EU directive. Mice were observed for clinical signs of tumor spreading and were killed if they developed >20% weight loss, hunched posture, ruffled fur, limb paralysis or enlarged spleens. Maximum tumor burden was not exceeded. Experiments were terminated 2 months after T cell injection to avoid graft-versus-host disease, and surviving mice were killed humanely by cervical dislocation. Six mice were housed per cage in Eurostandard Type III cages (macrolone) with a light cycle from 7 a.m. to 7 p.m. at 22 ± 1 °C with 62 ± 5% humidity. Female (8–10-week-old) NSG (Jackson Laboratory) mice, bred in house, were sublethally irradiated (2.5 Gy, MultiRad225 X-ray, RPS Services) on day −15, and 4 × 10^6^ cells of the human B-ALL cell line BV173^D835Y^ were injected on day −14 through the tail vein. After leukemia was confirmed by BLI on day −1, mice were treated with 10^7^ T cells transduced to express TCR^1G4^ or TCR^FLT3D/Y^. A group of control mice did not receive T cell injections. All mice were injected intraperitoneally (i.p.) daily with 2,500 IU IL-2 (R&D Systems). BLI imaging (by the IVIS Spectrum in vivo imaging system; analysis with Living Image software version 4.5.2, PerkinElmer) and blood analysis by flow cytometry were performed continuously. BM was collected at the endpoint and processed for flow cytometry to analyze the presence of T cells and tumor cells.

### Activity of TCR^FLT3D/Y^ cells in four primary AML PDX models

Experiments were approved by Stockholms Djurförsöksetiska nämnd (17978-2018). The maximal tumor burden permitted was defined by the impact on the animal’s health. Mice engrafted with leukemic cells were continuously monitored according to Karolinska Institutet’s health assessment, and no animal exceeded the humane endpoint. The housing conditions were 21 °C and 45–50% humidity. Two to five mice were housed per cage in IVC-Mouse GM500 cages with a light cycle from 4 a.m. to 4 p.m. (patient 7) or from 6 a.m. to 6 p.m. (patient 1). BM was collected from femur, tibia and crista from both hind legs at terminal analysis. Mainly female mice were used in this study, but, when both sexes were used, they were equally distributed within the treatment groups. No sex-based analysis was thus performed.

#### Model 1

Female NOD.Cg-*Prkdc*^scid^ *Il2rg*^*tm1Wjl*^ Tg(CMV IL3,CSF2,KITLG)1Eav/MloySzJ (NSG-SGM3) mice stably engrafted with FLT3^D835Y^-expressing HLA-A2^+^ AML cells from patient 7 (Supplementary Table 2 and 3) at 5 weeks of age were obtained from Jackson Laboratory (stock ID J000106565). Upon arrival (5.5 weeks after transplantation), PB myeloid engraftment was confirmed by flow cytometry, and mice were allocated to treatment groups (TCR^FLT3D/Y^ or TCR^1G4^ cells), resulting in a similar mean human myeloid engraftment between the groups before infusion with T cells. Cryopreserved T cells transduced to express TCR were thawed and cultured in X-VIVO 20 medium (Lonza) with 5% HS, 1% penicillin–streptavidin and 5 ng ml^−1^ IL-7 and IL-15 for 3–4 d before treatment. T cells containing 5 × 10^6^ CD8^+^mTCR-β^+^ T cells were injected through the lateral tail vein, and all mice received daily i.p. injections of 2,500 IU human IL-2 (R&D Systems). At day 8 after T cell infusion, half of the mice received a second dose of 5 × 10^6^ CD4-depleted (Miltenyi Biotec) TCR^FLT3D/Y^ or TCR^1G4^ T cells. As no differences were observed between the mice receiving one or two doses of T cells, the data from these mice were pooled. The effect of the T cells was monitored in serially collected PB and at termination 15 d after T cell treatment in the BM and spleen through detailed flow cytometry analysis.

#### Model 2

BM CD34^+^ cells from patient 1 (FLT3^D/Y^ and HLA-A2^+^; Supplementary Tables 2 and 3) were obtained by CD34 magnetic bead enrichment (Miltenyi Biotec) according to the manufacturer’s instructions and as previously described^78^. A total of 3 × 10^5^ CD34^+^ cells per mouse were intrafemorally injected into sublethally irradiated (3.3 Gy, X-ray source) female and male NSG-SGM3 mice (Jackson Laboratory, stock 013062) 9 weeks of age. Upon confirmation of stable PB myeloid engraftment 7 weeks after transplantation, mice were allocated to treatment groups (TCR^FLT3D/Y^ or TCR^1G4^ cells) based on their engraftment levels as described for patient 7. T cells containing 5 × 10^6^ CD8^+^mTCR-β^+^ T cells were infused into each mouse by lateral tail vain injections, and all mice received daily i.p. injections of 2,500 IU human IL-2 for 2 weeks, followed by less frequent injections. Mice were monitored using serially collected PB and at termination 34 d after T cell treatment with the BM and spleen.

#### Model 3

To mimic an MRD setting, secondary intrafemoral transplantation of BM cells from NSG mice (Jackson Laboratory, stock 005557) engrafted with material from patient 1 was performed into sublethally irradiated (2.5 Gy, X-ray source) female NSG mice 12–13 weeks of age. Following confirmation of low but stable leukemic engraftment 20 weeks after transplantation, mice were treated with TCR^FLT3D/Y^ or TCR^1G4^ CD4-depleted (Miltenyi Biotec) T cells (10 × 10^6^ T cells, containing 90% CD8^+^ and 10% CD4^+^ T cells). All mice were injected i.p. daily with 2,500 IU human IL-2. The effect of the T cells was evaluated in the BM 11 d after T cell treatment.

#### Model 4

Following secondary transplantation of BM from mice engrafted with patient 1 cells, BM from three NOD.Cg-*Prkdc*^scid^ *Il2rg*^*tm1Sug*^ Tg(CMV-IL2)4-2Jic/JicTac (NOG-hIL2, Taconic) mice was isolated and cultured for 48 h in CellGro GMP DC medium with 5% HS, IL-7 and IL-15 either with no T cells or with TCR^1G4^ or TCR^FLT3D/Y^ cells (E:T cell ratio, 1:2). After 48 h, the contents of each well were collected and intrafemorally injected into sublethally irradiated (2.25–2.5 Gy) female NSG mice 8–13 weeks of age. Engraftment levels were monitored in serially collected PB samples by flow cytometry. Data were pooled from two individual experiments. Differences in engraftment dynamics between mice engrafted with AML cells precultured with TCR^FLT3D/Y^ or TCR^1G4^ cells or without T cells was assessed by multilevel linear regression using the R package ‘lmerTest’. Sampling time points and groups are treated as an interaction term with random effects for individual mice. Because the engraftment at the first time point was 0 for all mice except one, we fixed the intercept as 0. Estimated marginal means of models were obtained and compared between three groups using the R package ‘emmeans’. Multiple tests were corrected using the Benjamini–Hochberg method. Only mice that could be followed until the end of the experiment were included in the analysis.

### Whole-exome sequencing of cells from patient 1 and PDX mice

DNA was isolated from AML cells purified with the FACSAria Fusion cell sorter (BD Biosciences) from patient 1 using the Maxwell RSC Cultured Cells DNA Kit (Promega), including primary BM AML blasts (CD3^−^CD19^−^) and T cells (CD3^+^CD8^+^CD19^−^CD33^−^ and CD3^+^CD4^+^CD19^-^CD33^-^) and BM mCD45^−^hCD45^+^CD3^−^CD19^−^ cells from one untreated and one TCR^1G4^-treated PDX mouse from model 2. Whole-exome sequencing libraries were prepared with the Lotus DNA Library Prep Kit (IDT). Exon regions were captured using xGen Exome Hyb Panel v2 and the xGen Hybridization and Wash Kit (IDT) on 4 July 2022 and 5 July 2022. After mixing with 1% PhiX, prepared libraries were sequenced using the NextSeq high-output kit (300 cycles) with settings ‘Read1, 151; Index1, 8; Index2, 8; Read2, 151’. Reads were aligned to the human (GRCh37) genome reference using Burrows–Wheeler Aligner version 0.7.17 with default parameter settings. PCR duplicates were marked with biobambam version 2.0.87. Reads were subjected to indel realignment and base quality score recalibration using GATK3 (version 3.8) and recalculation of MD/NM tags using SAMtools version 1.9. Errors associated with enzymatic fragmentation during library preparation were removed using FADE version 0.5.5, resulting in an average depth of 364 (331–439) in the final bam files^79^.

Mutations calling was performed using GenomonFisher (https://github.com/Genomon-Project/GenomonFisher) with the following parameters: (1) mapping quality score ≥20, (2) base quality score ≥15, (3) number of total reads ≥8, (4) number of variant reads ≥5, (5) VAF ≥ 0.05, (6) VAF in paired T cells <0.1, (7) VAF in other non-paired normal controls <0.05 in all and average <0.01, (8) strand ratio in tumor ≠0 or 1, (9) VAF by base counts divided by VAF by read count ≥0.5 and ≤2, (10) *P* value by Fisher <0.1, (11) *P* value by EBFilter^80^ <0.001, (12) mutation on exons, (13) not on the repeat regions.

Mutations were annotated by ANNOVAR^81^. Copy number analysis was performed using CNACS^82^. The WT1^H507P^ mutation was, together with the FLT3^D835Y^ mutation, the only identified potential driver mutation in AML^15,83^.

### Droplet digital PCR

ddPCR was performed to quantify clonal involvement. DNA from patient 1 with AML was isolated as explained above, and DNA from AML PDX mice was isolated through flow cytometry sorting of mCD45^−^mTer119^−^hCD45^+^CD3^−^ cells (PDX patient 7) or hCD45^+^CD33^+^CD34^−^, hCD45^+^CD33^+^CD34^+^ and hCD45^+^CD19^+^ cells (PDX patient 1) and subjected to whole-genome DNA amplification using the REPLI-g Single Cell Kit (QIAGEN) according to the manufacturer’s instructions. Samples with <90 cells were excluded. Briefly, a 20-µl PCR reaction mixture containing 1× ddPCR supermix for probes (no dUTP) (Bio-Rad), 1× primer-probe assay (FLT3^D835Y^, dHsaMDV2010047; WT1^H507P^, dHsaMDS871718945; Bio-Rad) and 60 ng DNA was mixed with Droplet Generation Oil for Probes (Bio-Rad). Droplets were prepared according to the manufacturer’s instructions on a QX200 droplet generator (Bio-Rad) and subjected to PCR (Bio-Rad): 95 °C for 10 min, 40 cycles of 94 °C for 30 s and 55 °C for 60 s and a 10-min incubation at 98 °C. Plates were read on a QX200 droplet reader (Bio-Rad) and analyzed using QuantaSoft version 1.5.38.1118 software (Bio-Rad) to calculate VAFs and 95% confidential intervals. The numbers of FLT3^D835Y^ cells in the AML PDX mice were determined by combining the information from the fraction of mutated cells identified by ddPCR, and the frequency of phenotypically defined subsets (hCD45^+^CD3^−^ or hCD45^+^CD33^+^CD34^−^ and hCD45^+^CD33^+^CD34^+^, respectively, for patients 7 and 1) in the BM as determined by flow cytometry, and the total number of MNCs in the BM was counted with Sysmex.

### External AML-targeted sequencing data analysis

Mutation data (SNVs and indels) from AML patients reported by Papaemmanuil et al.^15^ were downloaded from https://www.cbioportal.org. VAF was estimated from reported alternative allele reads divided by sequencing depth for the position. Patients harboring a FLT3^D835Y^ mutation were selected for in-depth analysis.

The order of FLT3 mutations in AML using VAF and the mutated cell fraction was determined from the publicly available mutation list identified by targeted DNA sequencing by Morita et al.^45^.

### Statistical analysis and reproducibility

Statistical analysis was performed in GraphPad Prism versions 6–8 (GraphPad Software). Comparison of mean values between two experimental groups was conducted with unpaired, two-tailed Student’s test. The ordinary ANOVA test with adjustment for multiple comparisons with Tukey’s post hoc test was employed for comparisons of more than two experimental groups. To determine differences between in vivo treatment groups in the PDX mouse models, Kruskal–Wallis ANOVA with Dunn’s multiple-comparison test and two-tailed Mann–Whitney test were performed. *P* values < 0.05 were considered statistically significant. The investigators were not blinded to allocation during experiments or to outcome assessment. PDX models 1–3 were performed once each, and data for PDX model 4 were generated from two independent experiments with mice from all treatment groups represented in both experiments. No statistical method was used to predetermine sample size, and experiments were not randomized. Sample sizes were estimated based on preliminary experiments and are similar to those previously reported^27,84^. Due to the nature of the other in vitro experiments, blinding was not possible and is not generally performed in the field as the data acquisition is quantitative (flow cytometry or MS) rather than qualitative and therefore less influenced by observer bias. Data distribution was assumed to be normal, but this was not formally tested. Data distribution is shown as individual data points.

### Illustrations

Fig. 1a was generated by Science Shaped (https://scienceshaped.com/). All mouse illustrations were generated using Adobe Illustrator 2022 version 26.0.3.

### Reporting summary

Further information on research design is available in the Nature Portfolio Reporting Summary linked to this article.

### Supplementary information

Supplementary Information Supplementary Tables 1–10. Supplementary Table 1: List of candidate off-target peptides reactive to TCRFLT3D/Y cells.Amino acid sequence of all potentially cross-reactive peptides predicted by the ScanProsite algorithm based on positive reactivities of the TCRFLT3D/Y cells against the peptide mimotope library of 161 peptides, shown in Fig. 1h. Supplementary Table 2: Clinical information for all leukemia patients included in the study.M, male; F, female; AML, acute myeloid leukemia; AMML, acute myelomonocytic leukemia; APL, acute promyelocytic leukemia. Cytogenetic characteristics from clinical diagnostics: TKD, tyrosine kinase domain; CFBbeta-MYH11 fusion inv(16)(p13,q22) is an inversion of chromosome 1–6, which results in a fused transcript between MYH11 (16p13.1) and CBFB (16q22.1); ITD, Internal tandem duplication; del(6q), deletion in long arm of chromosome 6. Allo-HSCT; allogeneic hematopoietic stem cell transplantation. Supplementary Table 3: Sequencing data for all FLT3^D/Y^ positive leukemia patients.PT ID, Patient ID; Chr = chromosome; VAF, Variant allele frequency. Data for patients 16 was acquired using the TruSight™ Myeloid Panel. Data for patient 7 was acquired using the DFCI Rapid Heme Panel v2.0 (JAX Laboratories). Data for patient 8 was acquired by a custom-designed targeted DNA sequencing panel^86^. Supplementary Table 4: Number of events recorded by flow cytometry in PB, BM and spleen at terminal analysis for PDX models.Total event count is defined as single, viable, MNCs (mCD45^+^ cells + hCD45^+^ cells) adjusted for T cells by subtraction of hCD3^+^ events. Supplementary Table 5: ddPCR analysis of the FLT3 D835Y mutation in BM from micePD–X model with AML patient 7.Mouse ID; Population; Treatment; Number of cells analyzed; %VAF, variant allele frequency; CI, confidence interval; min, minimum %VAF of 95% CI; max, maximum %VAF of 95% CI; %CD33^+^ cells of hCD45^+^CD3^-^ cells. Supplementary Table 6: Number of events recorded by flow cytometry in PB, BM and spleen at terminal analysis for PDX models.Total event count is defined as single, viable, MNCs (mCD45^+^ cells + hCD45^+^ cells) adjusted for T cells by subtraction of hCD3^+^ events. Supplementary Table 7: Somatic mutations in AML patient 1 BM blasts identified by whole exome sequencing.Somatic mutations identified by whole exome sequencing of blasts (CD3^−^CD19^−^) from BM of patient 1 were also detected in the two analyzed PDX mice transplanted with patient 1 blasts (one TCR1G4 cell treated mouse and one untreated mouse, both mCD45^−^hCD45+CD3^−^CD19). The FLT3D/Y mutation and a WT1 mutation (bold) were selected for further ddPCR analysis, shown in Fig. 4d and Extended Data Fig. 9c. Supplementary Table 8: ddPCR analysis of FLT3 D835Y and WT1 H507P mutations in BM from AML patient 1 and in transplanted PDX mice.Sample ID; Abbreviations; VAF, variant allele frequency; CI, confidence interval; min, mimimum VAF of 95% CI; max, maximum VAF of 95% CI. *Not enough cells for quantification. Supplementary Table 9: Number of events recorded by flow cytometry in PB, BM and spleen at terminal analysis for PDX models.Total event count is defined as single, viable, MNCs (mCD45^+^ cells + hCD45^+^ cells) adjusted for T cells by subtraction of hCD3^+^ events. Supplementary Table 10: Complete list of all antibodies used in the study.

Reporting Summary

### Source data

Source Data Fig. 1 Statistical source data for Fig. 1.

Source Data Fig. 2 Statistical source data for Fig. 2.

Source Data Fig. 3 Statistical source data for Fig. 3.

Source Data Fig. 4 Statistical source data for Fig. 4.

Source Data Fig. 5 Statistical source data for Fig. 5.

Source Data Extended Data Fig. 1 Statistical source data for Extended Data Fig. 1.

Source Data Extended Data Fig. 3 Statistical source data for Extended Data Fig. 3.

Source Data Extended Data Fig. 4 Statistical source data for Extended Data Fig. 4.

Source Data Extended Data Fig. 6 Statistical source data for Extended Data Fig. 6.

Source Data Extended Data Fig. 7 Statistical source data for Extended Data Fig. 7.

Source Data Extended Data Fig. 8 Statistical source data for Extended Data Fig. 8.

Source Data Extended Data Fig. 9 Statistical source data for Extended Data Fig. 9.

## Data Availability

The data that support the findings of this study are included in the text and in the Supplementary Information. Additional datasets used in the study are: the UniProt *Homo sapiens* database, using Mascot version 2.2.07 (https://www.matrixscience.com), curated human proteome databases UniProtKB/Swiss-Prot and the Protein Data Bank using the ScanProsite tool (https://prosite.expasy.org/scanprosite/), the pMHC class I binding prediction algorithm NetMHC version 4.0 (http://www.cbs.dtu.dk/services/NetMHC/), data from Papaemmanuil et al.^15^ (https://www.cbioportal.org) and data from Morita et al.^45^. Exome sequencing data have been deposited at the European Genome–Phenome Archive, which is hosted by the EBI and the CRG, under accession number EGAS00001007467 and can be shared according to institutional guidelines at Oslo University Hospital upon reasonable request. Mass spectrometry data have been deposited at the Proteomics Identification Database under accession number PXD043908. All other data supporting the findings of this study are available from the corresponding author on reasonable request. Source data are provided with this paper.

## References

[1] Coulie PG (1995). A mutated intron sequence codes for an antigenic peptide recognized by cytolytic T lymphocytes on a human melanoma. Proc. Natl Acad. Sci. USA.

[2] Wolfel T (1995). A p16INK4a-insensitive CDK4 mutant targeted by cytolytic T lymphocytes in a human melanoma. Science.

[3] Matsushita H (2012). Cancer exome analysis reveals a T-cell-dependent mechanism of cancer immunoediting. Nature.

[4] Castle JC (2012). Exploiting the mutanome for tumor vaccination. Cancer Res..

[5] van Rooij N (2013). Tumor exome analysis reveals neoantigen-specific T-cell reactivity in an ipilimumab-responsive melanoma. J. Clin. Oncol..

[6] Rizvi NA (2015). Cancer immunology. Mutational landscape determines sensitivity to PD-1 blockade in non-small cell lung cancer. Science.

[7] Yarchoan M, Hopkins A, Jaffee EM (2017). Tumor mutational burden and response rate to PD-1 inhibition. N. Engl. J. Med..

[8] Tran E (2014). Cancer immunotherapy based on mutation-specific CD4^+^ T cells in a patient with epithelial cancer. Science.

[9] Tran E (2016). T-cell transfer therapy targeting mutant KRAS in cancer. N. Engl. J. Med..

[10] Zacharakis N (2018). Immune recognition of somatic mutations leading to complete durable regression in metastatic breast cancer. Nat. Med..

[11] Karpanen T, Olweus J (2017). The potential of donor T-cell repertoires in neoantigen-targeted cancer immunotherapy. Front. Immunol..

[12] Foy SP (2023). Non-viral precision T cell receptor replacement for personalized cell therapy. Nature.

[13] Leidner R (2022). Neoantigen T-cell receptor gene therapy in pancreatic cancer. N. Engl. J. Med..

[14] Kim SP (2022). Adoptive cellular therapy with autologous tumor-infiltrating lymphocytes and T-cell receptor-engineered T cells targeting common p53 neoantigens in human solid tumors. Cancer Immunol. Res..

[15] Papaemmanuil E (2016). Genomic classification and prognosis in acute myeloid leukemia. N. Engl. J. Med..

[16] Socie G, Blazar BR (2009). Acute graft-versus-host disease: from the bench to the bedside. Blood.

[17] Mardiana S, Gill S (2020). CAR T cells for acute myeloid leukemia: state of the art and future directions. Front. Oncol..

[18] Daver N, Alotaibi AS, Bucklein V, Subklewe M (2021). T-cell-based immunotherapy of acute myeloid leukemia: current concepts and future developments. Leukemia.

[19] Kenderian SS (2015). CD33-specific chimeric antigen receptor T cells exhibit potent preclinical activity against human acute myeloid leukemia. Leukemia.

[20] Gill S (2014). Preclinical targeting of human acute myeloid leukemia and myeloablation using chimeric antigen receptor-modified T cells. Blood.

[21] Jetani H (2018). CAR T-cells targeting FLT3 have potent activity against FLT3^−^ITD^+^ AML and act synergistically with the FLT3-inhibitor crenolanib. Leukemia.

[22] Olweus J (1997). Dendritic cell ontogeny: a human dendritic cell lineage of myeloid origin. Proc. Natl Acad. Sci. USA.

[23] Kim MY (2018). Genetic inactivation of CD33 in hematopoietic stem cells to enable CAR T cell immunotherapy for acute myeloid leukemia. Cell.

[24] Haubner S (2019). Coexpression profile of leukemic stem cell markers for combinatorial targeted therapy in AML. Leukemia.

[25] Tambaro FP (2021). Autologous CD33-CAR-T cells for treatment of relapsed/refractory acute myelogenous leukemia. Leukemia.

[26] Laszlo GS, Estey EH, Walter RB (2014). The past and future of CD33 as therapeutic target in acute myeloid leukemia. Blood Rev..

[27] Ali, M. et al. T cells targeted to TdT kill leukemic lymphoblasts while sparing normal lymphocytes. *Nat. Biotechnol.* **40**, 488–498 (2021). 10.1038/s41587-021-01089-x PMC9005346 34873326

[28] Chapuis AG (2019). T cell receptor gene therapy targeting WT1 prevents acute myeloid leukemia relapse post-transplant. Nat. Med..

[29] van der Lee DI (2019). Mutated nucleophosmin 1 as immunotherapy target in acute myeloid leukemia. J. Clin. Invest..

[30] Xie G (2021). CAR-T cells targeting a nucleophosmin neoepitope exhibit potent specific activity in mouse models of acute myeloid leukaemia. Nat. Biomed. Eng..

[31] Biernacki MA (2020). CBFB–MYH11 fusion neoantigen enables T cell recognition and killing of acute myeloid leukemia. J. Clin. Invest..

[32] Daver N, Schlenk RF, Russell NH, Levis MJ (2019). Targeting *FLT3* mutations in AML: review of current knowledge and evidence. Leukemia.

[33] Daver N (2015). Secondary mutations as mediators of resistance to targeted therapy in leukemia. Blood.

[34] Stone RM (2017). Midostaurin plus chemotherapy for acute myeloid leukemia with a *FLT3* mutation. N. Engl. J. Med..

[35] Smith CC (2012). Validation of ITD mutations in FLT3 as a therapeutic target in human acute myeloid leukaemia. Nature.

[36] Smith CC, Lin K, Stecula A, Sali A, Shah NP (2015). FLT3 D835 mutations confer differential resistance to type II FLT3 inhibitors. Leukemia.

[37] Smith CC (2017). Heterogeneous resistance to quizartinib in acute myeloid leukemia revealed by single-cell analysis. Blood.

[38] Stronen E (2016). Targeting of cancer neoantigens with donor-derived T cell receptor repertoires. Science.

[39] Ali M (2019). Induction of neoantigen-reactive T cells from healthy donors. Nat. Protoc..

[40] Cohen CJ, Zhao Y, Zheng Z, Rosenberg SA, Morgan RA (2006). Enhanced antitumor activity of murine–human hybrid T-cell receptor (TCR) in human lymphocytes is associated with improved pairing and TCR/CD3 stability. Cancer Res..

[41] Robbins PF (2011). Tumor regression in patients with metastatic synovial cell sarcoma and melanoma using genetically engineered lymphocytes reactive with NY-ESO-1. J. Clin. Oncol..

[42] Bethune MT (2018). Isolation and characterization of NY-ESO-1-specific T cell receptors restricted on various MHC molecules. Proc. Natl Acad. Sci. USA.

[43] Marcu A (2021). HLA Ligand Atlas: a benign reference of HLA-presented peptides to improve T-cell-based cancer immunotherapy. J. Immunother. Cancer.

[44] Jaiswal S, Ebert BL (2019). Clonal hematopoiesis in human aging and disease. Science.

[45] Morita K (2020). Clonal evolution of acute myeloid leukemia revealed by high-throughput single-cell genomics. Nat. Commun..

[46] Shlush LI (2014). Identification of pre-leukaemic haematopoietic stem cells in acute leukaemia. Nature.

[47] Goardon N (2011). Coexistence of LMPP-like and GMP-like leukemia stem cells in acute myeloid leukemia. Cancer Cell.

[48] Lapidot T (1994). A cell initiating human acute myeloid leukaemia after transplantation into SCID mice. Nature.

[49] Bonnet D, Dick JE (1997). Human acute myeloid leukemia is organized as a hierarchy that originates from a primitive hematopoietic cell. Nat. Med..

[50] Schuurhuis GJ (2018). Minimal/measurable residual disease in AML: a consensus document from the European LeukemiaNet MRD Working Party. Blood.

[51] Pearlman AH (2021). Targeting public neoantigens for cancer immunotherapy. Nat. Cancer.

[52] Bassani-Sternberg M, Coukos G (2016). Mass spectrometry-based antigen discovery for cancer immunotherapy. Curr. Opin. Immunol..

[53] Loffler MW (2019). Multi-omics discovery of exome-derived neoantigens in hepatocellular carcinoma. Genome Med..

[54] Sykulev Y, Joo M, Vturina I, Tsomides TJ, Eisen HN (1996). Evidence that a single peptide–MHC complex on a target cell can elicit a cytolytic T cell response. Immunity.

[55] Scheper W (2019). Low and variable tumor reactivity of the intratumoral TCR repertoire in human cancers. Nat. Med..

[56] Lowery FJ (2022). Molecular signatures of antitumor neoantigen-reactive T cells from metastatic human cancers. Science.

[57] Nagarsheth NB (2021). TCR-engineered T cells targeting E7 for patients with metastatic HPV-associated epithelial cancers. Nat. Med..

[58] Sahin U (2017). Personalized RNA mutanome vaccines mobilize poly-specific therapeutic immunity against cancer. Nature.

[59] Ott PA (2017). An immunogenic personal neoantigen vaccine for patients with melanoma. Nature.

[60] Lang, F., Schrors, B., Lower, M., Tureci, O. & Sahin, U. Identification of neoantigens for individualized therapeutic cancer vaccines. *Nat. Rev. Drug Discov.* **21**, 261–282 (2022). 10.1038/s41573-021-00387-y PMC7612664 35105974

[61] Johnson LA (2009). Gene therapy with human and mouse T-cell receptors mediates cancer regression and targets normal tissues expressing cognate antigen. Blood.

[62] Jin BY (2018). Engineered T cells targeting E7 mediate regression of human papillomavirus cancers in a murine model. JCI Insight.

[63] Duan F (2014). Genomic and bioinformatic profiling of mutational neoepitopes reveals new rules to predict anticancer immunogenicity. J. Exp. Med..

[64] Chandran SS (2022). Immunogenicity and therapeutic targeting of a public neoantigen derived from mutated *PIK3CA*. Nat. Med..

[65] Foldvari Z, Knetter C, Yang W, et al. A systematic safety pipeline for selection of T-cell receptors to enter clinical use. *NPJ Vaccines*10.1038/s41541-023-00713-y (2023). 10.1038/s41541-023-00713-y PMC10444760 37607971

[66] Jan M (2012). Clonal evolution of preleukemic hematopoietic stem cells precedes human acute myeloid leukemia. Sci. Transl. Med..

[67] Mansilla-Soto J (2022). HLA-independent T cell receptors for targeting tumors with low antigen density. Nat. Med..

[68] Dolgin E (2022). First soluble TCR therapy opens ‘new universe’ of cancer targets. Nat. Biotechnol..

[69] Abrahamsen IW (2010). Targeting B cell leukemia with highly specific allogeneic T cells with a public recognition motif. Leukemia.

[70] Kumari S (2014). Alloreactive cytotoxic T cells provide means to decipher the immunopeptidome and reveal a plethora of tumor-associated self-epitopes. Proc. Natl Acad. Sci. USA.

[71] Toebes M (2006). Design and use of conditional MHC class I ligands. Nat. Med..

[72] Hadrup SR (2009). Parallel detection of antigen-specific T-cell responses by multidimensional encoding of MHC multimers. Nat. Methods.

[73] Linnemann C (2013). High-throughput identification of antigen-specific TCRs by TCR gene capture. Nat. Med..

[74] Brochet X, Lefranc MP, Giudicelli V (2008). IMGT/V-QUEST: the highly customized and integrated system for IG and TR standardized V-J and V-D-J sequence analysis. Nucleic Acids Res..

[75] Toebes, M., Rodenko, B., Ovaa, H. & Schumacher, T. N. Generation of peptide MHC class I monomers and multimers through ligand exchange. *Curr. Protoc. Immunol.* **87**, 18.16.1–18.16.20 (2009). 10.1002/0471142735.im1816s87 19918947

[76] Sikorski K (2018). A high-throughput pipeline for validation of antibodies. Nat. Methods.

[77] Carrelha J (2018). Hierarchically related lineage-restricted fates of multipotent haematopoietic stem cells. Nature.

[78] Woll PS (2014). Myelodysplastic syndromes are propagated by rare and distinct human cancer stem cells in vivo. Cancer Cell.

[79] Gregory T (2020). Characterization and mitigation of fragmentation enzyme-induced dual stranded artifacts. NAR Genom. Bioinform..

[80] Shiraishi Y (2013). An empirical Bayesian framework for somatic mutation detection from cancer genome sequencing data. Nucleic Acids Res..

[81] Wang K, Li M, Hakonarson H (2010). ANNOVAR: functional annotation of genetic variants from high-throughput sequencing data. Nucleic Acids Res..

[82] Yoshizato T (2017). Genetic abnormalities in myelodysplasia and secondary acute myeloid leukemia: impact on outcome of stem cell transplantation. Blood.

[83] Rampal R, Figueroa ME (2016). Wilms tumor 1 mutations in the pathogenesis of acute myeloid leukemia. Haematologica.

[84] Jain N (2023). TET2 guards against unchecked BATF3-induced CAR T cell expansion. Nature.

[85] Jurtz V (2017). NetMHCpan-4.0: improved peptide–MHC class I interaction predictions integrating eluted ligand and peptide binding affinity data. J. Immunol..

[86] Wang M (2017). Validation of risk stratification models in acute myeloid leukemia using sequencing-based molecular profiling. Leukemia.

