# Supplementary material for: A T cell receptor targeting a recurrent driver mutation in FLT3 mediates elimination of primary human acute myeloid leukemia in vivo
Source: Nat Cancer. 2023 Oct 2;4(10):1474–90. doi: 10.1038/s43018-023-00642-8 (PMC10597840; doi:10.1038/s43018-023-00642-8)
Supplement: Supplementary file 1 — Supplementary Tables 1–10. Supplementary Table 1: List of candidate off-target peptides reactive to TCRFLT3D/Y cells.Amino acid sequence of all potentially cross-reactive peptides predicted by the ScanProsite algorithm based on positive reactivities of the TCRFLT3D/Y cells against the peptide mimotope library of 161 peptides, shown in Fig. 1h. Supplementary Table 2: Clinical information for all leukemia patients included in the study.M, male; F, female; AML, acute myeloid leukemia; AMML, acute myelomonocytic leukemia; APL, acute promyelocytic leukemia. Cytogenetic characteristics from clinical diagnostics: TKD, tyrosine kinase domain; CFBbeta-MYH11 fusion inv(16)(p13,q22) is an inversion of chromosome 1–6, which results in a fused transcript between MYH11 (16p13.1) and CBFB (16q22.1); ITD, Internal tandem duplication; del(6q), deletion in long arm of chromosome 6. Allo-HSCT; allogeneic hematopoietic stem cell transplantation. Supplementary Table 3: Sequencing data for all FLT3D/Y positive leukemia patients.PT ID, Patient ID; Chr = chromosome; VAF, Variant allele frequency. Data for patients 16 was acquired using the TruSight™ Myeloid Panel. Data for patient 7 was acquired using the DFCI Rapid Heme Panel v2.0 (JAX Laboratories). Data for patient 8 was acquired by a custom-designed targeted DNA sequencing panel86. Supplementary Table 4: Number of events recorded by flow cytometry in PB, BM and spleen at terminal analysis for PDX models.Total event count is defined as single, viable, MNCs (mCD45+ cells + hCD45+ cells) adjusted for T cells by subtraction of hCD3+ events. Supplementary Table 5: ddPCR analysis of the FLT3 D835Y mutation in BM from micePD–X model with AML patient 7.Mouse ID; Population; Treatment; Number of cells analyzed; %VAF, variant allele frequency; CI, confidence interval; min, minimum %VAF of 95% CI; max, maximum %VAF of 95% CI; %CD33+ cells of hCD45+CD3- cells. Supplementary Table 6: Number of events recorded by flow cytometry in PB, BM and spleen [file 43018_2023_642_MOESM1_ESM.pdf]

nature cancer

Article

https://doi.org/10.1038/s43018-023-00642-8

A T cell receptor targeting a recurrent driver mutation in FLT3 mediates elimination of primary human acute myeloid leukemia in vivo

In the format provided by the authors and unedited

Supplementary Table S1:List of candidate off-target peptides reactive to TCR$^{FLT3D/Y}$ cells

Peptide Number	Peptide Sequence	Gene Name	Full Name
1	TMINDSDYV	*ABCA5*	Cholesterol transporter ABCA5
2	FLKNDMIYA	*AP5M1*	AP-5 complex subunit mu-1
3	SLISDSAYV	*ASCC3*	Activating signal cointegrator 1 complex subunit 3
4	YTGTDTQYV	*CADH9*	Cadherin-9
5	ITINDVVYV	*DHX9*	ATP-dependent RNA helicase A
6	STQTDIFYT	*DLG5*	Disks large homolog 5
7	GVGTDTNYV	*DLGP4*	Disks large-associated protein 4 (DAP-4)
8	AMMCDIIYA	*ECHM*	Enoyl-CoA hydratase, mitochondrial (mECH)
9	SINNDTTYC	*LACB2*	Endoribonuclease LACTB2
10	QIYTDIIYI	*LFG3*	Protein lifeguard 3
11	AVISDAMYI	*LZTR1*	Leucine-zipper-like transcriptional regulator 1
12	YITSDMFYV	*MED1*	Mediator of RNA polymerase II transcription subunit 1
13	FLSNDAAYT	*MMP15*	Matrix metalloproteinase-15
14	VLTSDMAYV	*P3C2A*	Phosphatidylinositol 4-phosphate 3-kinase C2 domain-containing subunit alpha
15	AVDNDSFYV	*PRADC1*	Protease-associated domain-containing protein 1
16	FVDSDSTYC	*PASD1*	Circadian clock protein PASD1
17	SVSTDTIYA	*PIGC*	Phosphatidylinositol N-acetylglucosaminyltransferase subunit C
18	MVQTDMQYV	*PTPRA*	Receptor-type tyrosine-protein phosphatase alpha
19	MVQTDMQYT	*PTPRE*	Receptor-type tyrosine-protein phosphatase epsilon
20	QTKSDSNYI	*SETMR*	Histone-lysine N-methyltransferase SETMAR
21	HLRNDSHYV	*SLC31*	Neutral and basic amino acid transport protein rBAT
22	LTTSDIDYA	*TAF6*	Transcription initiation factor TFIID subunit 6
23	HVWTDTHYV	*TFR2*	Transferrin receptor protein 2
24	ALSNDTVYV	*THSD1*	Thrombospondin type-1 domain-containing protein 1
25	LTCTDCGYC	*UBP2*	Ubiquitin carboxyl-terminal hydrolase 2
26	ALFTDTGYI	*VPS16*	Vacuolar protein sorting-associated protein 16 homolog
27	VTGTDSQYC	*WDR62*	WD repeat-containing protein
28	GLESDVAYA	*ZCPW1*	Zinc finger CW-type PWWP domain protein 1

Supplementary Table S2: Clinical information for all leukemia patients included in the study

PT ID	Sex and (Age)	Classification	Cytogenetic Characteristics	TKD^{mut} type	HLA*02:01 Status	Risk Stratification	Response to HSCT	Outcome
PT. 1	M (65)	AML	*FLT3* TKD *CBFbeta-MYH 11* fusion inv(16)(p13;q22)	D835Y	+	High Risk	Complete remission Allo-HSCT	Alive
PT. 2	M (20)	AMML	*FLT3* TKD	D835Y	+	High Risk	Complete remission Allo-HSCT	Alive
PT. 3	F (45)	AML	*FLT3* TKD *NPM1* mutation	D835Y	+	Low Risk	Complete remission Auto-HSCT	Alive
PT. 4	M (91)	AML	*FLT3* TKD	D835Y	+	Intermediate	No treatment	Dead
PT. 5	M (56)	APL	*FLT3* TKD, *FLT3*-ITD, *NRAS*	D835Y	+	Low Risk	Complete remission Not transplanted	Alive
PT. 6	F (72)	AML	*FLT3* TKD, *NPM1* mutation, *IDH1*, *DNMT3A*	D835Y	+	Intermediate	Palliative treatment with hydroxyurea	Dead
PT. 7	M (61)	AML	*FLT3* ITD, *FLT3* TKD, *NPM1,* Trisomy 8,Trisomy 14, del(6q)	D835Y	+	Intermediate	Residual AML Allo-HSCT	Not known
PT. 8	F (72)	AMML	*FLT3* TKD, *DNMT3A*, *NPM1*	D835Y	-	Low Risk	Residual AML	Dead
PT. 9	F (44)	AML	*FLT3* TKD *NPM1* mutation	D835E	+	High Risk	Residual AML Allo-HSCT	Dead
PT.10	F (33)	AMML	*FLT3* TKD *CBFbeta-MYH 11* fusion inv(16)(p13;q22)	D835H	+	Low Risk	Complete remission Not transplanted	Alive
PT.11	M (57)	AML	Trisomy 8 and *NPM1* mutation	-	+	Low Risk	Complete remission Allo-HSCT	Alive

Supplementary Table S3: Sequencing data for all FLT3$^{D/Y}$ positive leukemia patients

PT. ID	Chr	Gene	Type	Transcript Variant	Protein Varient	VAF(%)
Pt. 1	13	*FLT3*	missense/snv	c.2503G>T	p.D835Y	45,84
Pt. 2	13	*FLT3*	missense/snv	c.2503G>T	p.D835Y	47,14
Pt. 3	2<br>5<br><br>10<br>13	*DNMT3A*<br>*NPM1*<br><br>*SMC3*<br>*FLT3*	missense/snv<br>frameshift/insertion<br><br>missense/snv<br>missense/snv	c.2645G>A; n.3075G>A; c.2189G>A; c.2078G>A<br>c.773_776dupTCTG; n.977_980dupTCTG;<br>c.860_863dupTCTG; c.479_482dupTCTG;<br>c.668_671dupTCTG c.1142G>T n.2701G>T;<br>c.2503G>T	p.R693H; p.R882H; p.R730H<br>p.W161fs*?; p.W259fs*?; p.W288fs*?;<br><br>p.W224fs*? p.R381L<br>p.D835Y	41,8<br>53,3<br><br>30,4<br>38,9
Pt. 4	8<br>13<br>17<br><br><br><br>21<br>21<br>X<br>X	*RAD21*<br>*FLT3 MFSD11;*<br>*MIR636;*<br>*SRSF2*<br><br><br>*RUNX1*<br>*RUNX1*<br>*BCOR*<br>*BCORL1*	frameshift/deletion<br>missense/snv<br>missense/snv<br><br><br><br>missense/snv stop gain/snv<br>frameshift/insertion stop<br>gain/snv<br>missense/snv	c.355delA; c.1720delA; c.232delA; c.34delA<br>n.2701G>T; c.2503G>T<br>c.-1193G>C; n.-329C>G; c.-281G>C; c.- 1078G>C;<br>c.-817G>C; n.-624G>C; c.-286G>C; c.-1476G>C;<br>c.-1360G>C; c.-1171G>C; n.313G>C;<br>n.455C>G; c.284C>G<br>c.493G>T; c.412G>T<br>c.422C>A; c.341C>A<br>c.2811dupC<br>c.2350C>T	p.T119fs*38; p.T78fs*38; p.T12fs*38; p.T574fs*38<br>p.D835Y<br>p.P95R<br><br><br><br>p.G165C; p.G138C<br>p.S141*; p.S114*<br>p.T938fs*8<br>p.R784*	42,5<br>27,8<br>45,5<br><br><br><br>44,1<br>43,8<br>80,6<br>84,2
Pt. 5	7<br>13	*CUX1*<br>*FLT3*	missense/snv<br>missense/snv	 c.2503G>T	 p.D835Y	48,5<br>25,98
Pt. 6	2<br>2<br>5<br><br>13	*DNMT3A*<br>*IDH1*<br>*NPM1*<br><br>*FLT3*	missense/snv<br>missense/snv<br>frameshift/insertion<br><br>missense/snv	n.3074C>T; c.2644C>T; c.2077C>T; c.2188C>T<br>c.395G>A<br>c.776_777insCCTG; c.863_864insCCTG;<br>n.980_981insCCTG; c.482_483insCCTG;<br>c.671_672insCCTG<br>c.2503G>T	p.R693C; p.R882C; p.R730C<br>p.R132H<br>p.W161fs*?; p.W259fs*?; p.W288fs*?; p.W224fs*?<br><br>p.D835Y	48,3<br>47,2<br>43,5<br><br>46,2
Pt. 7	13<br>13<br><br>5	*FLT3*<br>*FLT3*<br><br>*NPM1*	missense/snv<br>frameshift/insertion<br><br>frameshift/insertion	c.2503C>A<br>28608259 sequence<br>AGCCAGCTACAGATGGTACAGGTGACCGGCTCCTCAGATAATGAGTA<br>CTCC<br>c.859_860insTCTG	p.D835Y<br><br><br>p.W288fs*>9	43<br><br><br>38,1
Pt. 8	2<br>5<br>13	*DNMT3A*<br>*NPM1*<br>*FLT3*	frameshift/deletion<br>frameshift/insertion<br>missence/snv	c.1521delC<br>c.858insTCTG<br>c.2503C>A	p.L508fs<br>p.L287fs<br>p.D835Y	29<br>30<br>44

Supplementary Table S4: Number of events recorded by flow cytometry in PB, BM and spleen at terminal analysis for PDX models

PDX model patient 7					
Mouse ID	Treatment	BM		Spleen	
		Total events	CD33+ events	Total events	CD33+ events
1	TCR^{1G4}	23069	20344	53485	3193
3	TCR^{1G4}	94332	90434	52402	5434
7	TCR^{1G4}	89313	83333	54036	6720
8	TCR^{1G4}	33993	21127	60046	3923
10	TCR^{1G4}	88895	87634	46640	10104
12	TCR^{1G4}	24153	21654	48277	2953
2	$TCR^{FLT3D/Y}$	62264	26	109031	3
4	$TCR^{FLT3D/Y}$	39702	151	95415	40
5	$TCR^{FLT3D/Y}$	45769	309	81054	8
6	$TCR^{FLT3D/Y}$	41736	445	121855	23
9	$TCR^{FLT3D/Y}$	21850	199	93328	20
11	$TCR^{FLT3D/Y}$	47639	12	77626	5
13	$TCR^{FLT3D/Y}$	13048	53	78465	6

Supplementary Table S5: ddPCR analysis of the FLT3 D835Y mutation in BM from mice - PDX model with AML patient 7

					95% CI		
**Mouse ID**	**Population**	**Treatment**	**Number of cells**	**%VAF**	**Min**	**Max**	**% CD33+ cells**
1	CD45+CD3-	TCR^{1G4}	2000	99.8	99.7	99.9	99.1
3	CD45+CD3-	TCR^{1G4}	2000	99.4	99.2	99.7	99.6
7	CD45+CD3-	TCR^{1G4}	2000	99.9	99.7	100	99.6
8	CD45+CD3-	TCR^{1G4}	2000	99	98.8	99.3	99.6
10	CD45+CD3-	TCR^{1G4}	2000	99.8	99.7	99.9	99.5
12	CD45+CD3-	TCR^{1G4}	2000	99.1	98.9	99.3	99.1
2	CD45+CD3-	$TCR^{FLT3D/Y}$	251	5.2	4.6	5.8	8.6
4	CD45+CD3-	$TCR^{FLT3D/Y}$	150	42.8	40.4	45.2	53
5	CD45+CD3-	$TCR^{FLT3D/Y}$	300	68.5	67.4	69.6	60.5
6	CD45+CD3-	$TCR^{FLT3D/Y}$	568	59.8	58.4	61.3	64.6
9	CD45+CD3-	$TCR^{FLT3D/Y}$	281	39.9	38.7	41.2	46.3
11	CD45+CD3-	$TCR^{FLT3D/Y}$	268	4.8	4.3	5.3	6
13	CD45+CD3-	$TCR^{FLT3D/Y}$	218	29.9	28.3	31.5	21.6

Calculations for Main Fig 3E: ((fraction of hCD45+CD3- cells) x (VAF/100) x cellularity in BM)
Determined by FACS, ddPCR and sysmex count respectively.

Supplementary Table S6: Number of events recorded by flow cytometry in PB, BM and spleen at terminal analysis for PDX models

PDX model patient 1													
Mouse ID	Treatment	PB				BM				Spleen			
		Total events	CD33+ events	CD33+CD34- events	CD33+CD34+ events	Total events	CD33+ events	CD33+CD34- events	CD33+CD34+ events	Total events	CD33+ events	CD33+CD34- events	CD33+CD34+ events
14	TCR^{1G4}	20387	915	831	84	81114	22366	17649	4717	42390	1400	1158	242
15	TCR^{1G4}	25398	1087	982	105	52782	15358	12775	2583	55286	1085	831	254
16	TCR^{1G4}	16848	2496	2269	227	91754	29039	24188	4851	58426	1283	1096	187
17	TCR^{1G4}	63157	191	180	11	85490	5629	4385	1244	76297	230	210	20
18	$TCR^{FLT3D/Y}$	13802	150	149	1	253829	2286	2273	13	64374	566	563	3
19	$TCR^{FLT3D/Y}$	**19237**	327	327	0	220258	4687	4680	7	87812	778	776	2
20	$TCR^{FLT3D/Y}$	8943	347	347	0	165092	3737	3733	4	36631	394	394	0
21	$TCR^{FLT3D/Y}$	16968	180	180	0	150235	2057	2049	8	36047	266	264	2

Supplementary Table S7:Somatic mutations in AML patient 1 BM blasts identified by whole exome sequencing

	Detected in sample (YES/NO)		
**Mutations detected in patient 1 BM blasts**	**Untreated PDX mouse**	**TCR[1G4] treated PDX mouse**	**Patient 1 BM T cells**
**FLT3:NM_004119:exon20:c.G2503T:p.D835Y**	YES	YES	NO
**WT1:NM_024426:exon10:c.A1520C:p.H507P**	YES	YES	NO
BSN:NM_003458:exon6:c.G10595C:p.C3532S	YES	YES	NO
ADAMTSL4:NM_019032:exon11:c.C1825A:p.P609T	YES	YES	NO
NOL4:NM_001353236:exon6:c.A870G:p.X290W	YES	YES	NO
FBXW12:NM_001159927:exon8:c.G869A:p.G290E	YES	YES	NO
TPH2:NM_173353:exon4:c.540+2T>G	YES	YES	NO
ZNF646:NM_014699:exon2:c.G5192A:p.R1731H	YES	YES	NO
ITGA2B:NM_000419:exon5:c.G608C:p.S203T	YES	YES	NO
HTT:NM_002111:exon25:c.G3145A:p.V1049M	YES	YES	NO
TTC21A:NM_001105513:exon2:c.C137A:p.A46D	YES	YES	NO
LPA:NM_005577:exon39:c.A5968T:p.S1990C	YES	YES	NO
SCN9A:NM_001365536:exon12:c.G1951T:p.D651Y	YES	YES	NO
ANHX:NM_001191054:exon7:c.C901G:p.L301V	YES	YES	NO
NWD1:NM_001007525:exon5:c.C361T:p.L121L	YES	YES	NO
SDR16C5:NM_001318050:exon6:c.C722G:p.T241R	YES	YES	NO
HAVCR2:NM_032782:exon7:c.C775T:p.R259C	YES	YES	NO
ZNF266:NM_001370390:exon8:c.G305A:p.S102N	YES	YES	NO
CCNJL:NM_001308173:exon6:c.C875T:p.A292V	YES	YES	NO
BTBD7:NM_001289133:exon5:c.A644G:p.N215S	YES	YES	NO
WNK2:NM_006648:exon22:c.C5646T:p.S1882S	YES	YES	NO
LTBR:NM_002342:exon1:c.A19C:p.T7P	YES	YES	NO
ARIH2:NM_001317334:exon5:c.C179A:p.P60H	YES	YES	NO

Supplementary Table S8:ddPCR analysis of FLT3 D835Y and WT1 H507P mutations in BM from AML patient 1 and in transplanted PDX mice

				FLT3 D835Y			WT1 H507P		
					95 % CI			95 % CI	
ID	Population	Treatment	Number of cells	%VAF	Min	Max	%VAF	Min	Max
Primary patient 1 BM blasts	CD3-CD19-	N/A	933 991	46,1	45,2	47,0	45,54	44,69	46,39
Mouse 14	CD33+CD34-	1G4	1000	43,3	42,0	44,7	42,3	41,3	43,3
	CD33+CD34+		1000	47,9	46,7	49,0	50,9	49,8	51,9
	CD19+		1000	0,4	0,2	0,5	0,4	0,3	0,5
Mouse 15	CD33+CD34-	1G4	1000	38,7	37,4	39,9	39,9	39,0	40,8
	CD33+CD34+		1000	49,2	48,0	50,5	51,1	50,2	52,0
	CD19+		1000	0,0	0,0	0,0	N/A	N/A	N/A
Mouse 16	CD33+CD34-	1G4	1000	42,0	40,7	43,2	42,8	41,8	43,8
	CD33+CD34+		1000	49,8	48,4	51,1	50,0	49,0	50,9
	CD19+		1000	0,0	0,0	0,0	N/A	N/A	N/A
Mouse 17	CD33+CD34-	1G4	1000	42,8	41,5	44,2	42,4	41,4	43,3
	CD33+CD34+		1000	50,7	49,4	52,1	50,2	49,2	51,2
	CD19+ *		10	0,0	0,0	0,1	N/A	N/A	N/A
Mouse 18	CD33+CD34-	FLT3 D/Y	1000	0,4	0,2	0,5	0,4	0,3	0,5
	CD19+		500	0,0	0,0	0,0	N/A	N/A	N/A
Mouse 19	CD33+CD34-	FLT3 D/Y	1000	0,2	0,1	0,3	0,4	0,3	0,5
	CD19+		1000	0,0	0,0	0,1	N/A	N/A	N/A
Mouse 20	CD33+CD34-	FLT3 D/Y	1000	0,8	0,6	1,1	0,3	0,2	0,4
	CD19+		659	0,0	0,0	0,0	N/A	N/A	N/A
Mouse 21	CD33+CD34-	FLT3 D/Y	1000	0,5	0,3	0,6	0,3	0,2	0,3
	CD19+		90	0,0	0,0	0,0	N/A	N/A	N/A

Calculations for Main Fig 4E: ((fraction of hCD45+CD33+CD34+/- cells) x (VAF/100) x cellularity in BM) Determined by FACS, ddPCR and sysmex count respectively.
*insufficient cells for quantification

Supplementary Table S9: Number of events recorded by flow cytometry in PB, BM and spleen at terminal analysis for PDX models

PDX model patient 1 MRD			
Mouse ID	Treatment	BM	
		Total events	CD33+ events
23	TCR^{1G4}	287927	495
25	TCR^{1G4}	195571	160
27	TCR^{1G4}	245213	266
30	TCR^{1G4}	251089	105
22	$TCR^{FLT3D/Y}$	250777	4
24	$TCR^{FLT3D/Y}$	301753	1
26	$TCR^{FLT3D/Y}$	247626	0
28	$TCR^{FLT3D/Y}$	4246	0

Supplementary Table S10: Complete list of all antibodies used in the study

Antibody List

CD number/protein target/dye/kit	Fluorochrome	Clone name	Species	Supplier	Catalogue number	Dilution factor	Application	Lot	Validation
CD3	PerCP-Cy5.5, APC-Cy7, BB515,	SK7, UCH-T1, HIT3a	Mouse, anti-human	Biolegend, Biolegend, BD	344808, 300426, 565100	1:200, 1:200, 1:100-1:200	Flow cytometry	(BB515) 7158825/ 9021562/ 9205788/ 0077790	https://www.biolegend.com/ja-jp/quality/quality-control<br>https://www.bdbiosciences.com/en-us/products/reagents/flow-cytometry-reagents/research-reagents/quality-and-reproducibility
CD4	BUV395, PE-Cy5, BV711	RPA-T4, OKT4	Mouse, anti-human	BD, Biolegend, Biolegend	564724, 300512, 317440	1:300, 1;300, 1:200	Flow cytometry	(BUV395) 35962, (PE-Cy7) B269744/ B288879	https://www.bdbiosciences.com/en-us/products/reagents/flow-cytometry-reagents/research-reagents/quality-and-reproducibility<br>https://www.biolegend.com/ja-jp/quality/quality-control
CD8a	AF700, BV421, APC-Cy7	HIT8a, RPA-T8	Mouse, anti-human	Biolegend	300920, 301036, 301016	1:200, 1:200, 1:80-1:300	Flow cytometry	(APC-Cy7) B274260/ B300873	https://www.biolegend.com/ja-jp/quality/quality-control
CD11b	FITC	ICRF44	Mouse, anti-human	Biolegend	301330	1:200	Flow cytometry		https://www.biolegend.com/ja-jp/quality/quality-control
CD13	BV711	WM15	Mouse, anti-human	Biolegend	301722	1:200	Flow cytometry		https://www.biolegend.com/ja-jp/quality/quality-control
CD14	FITC, BV711, PE-Cy7	HCD14, M5E2	Mouse, anti-human	Biolegend	325604, 301838, 301820	1:200	Flow cytometry		https://www.biolegend.com/ja-jp/quality/quality-control
CD16	FITC	3G8, NKP15	Mouse, anti-human	Biolegend, BD	302006, 335035	1:200, 1:100	Flow cytometry		https://www.biolegend.com/ja-jp/quality/quality-control<br>https://www.bdbiosciences.com/en-us/products/reagents/flow-cytometry-reagents/research-reagents/quality-and-reproducibility
CD19	BV785, BV421, FITC	HIB19, SJ25c1, 4G7),	Mouse, anti-human	Biolegend, Biolegend, BD	302240, 363018, 345776	1:200, 1:200-1:300, 1:100	Flow cytometry	(BV421) B237452/B313 190	https://www.biolegend.com/ja-jp/quality/quality-control<br>https://www.bdbiosciences.com/en-us/products/reagents/flow-cytometry-reagents/research-reagents/quality-and-reproducibility
CD20	BV785	2H7	Mouse, anti-human	Biolegend	302356	1:200	Flow cytometry		https://www.biolegend.com/ja-jp/quality/quality-control
CD33	APC-Cy7, BV785	P67.6, VM-53	Mouse, anti-human	Biolegend	366614, 303428	1:200	Flow cytometry	(BV785) B252804/ B260381/ B297609	https://www.biolegend.com/ja-jp/quality/quality-control
CD34	BV785, AF647, APC	561, 581, 8G12	Mouse, anti-human	Biolegend, Biolegend, BD	343626, 343508, 345804	1:200	Flow cytometry	(APC) 7348707	https://www.biolegend.com/ja-jp/quality/quality-control<br>https://www.bdbiosciences.com/en-us/products/reagents/flow-cytometry-reagents/research-reagents/quality-and-reproducibility
CD38	PE-Cy7	HB-7	Mouse, anti-human	Biolegend	356608	1:200	Flow cytometry		https://www.biolegend.com/ja-jp/quality/quality-control
CD45	BV605, AF700	H130	Mouse, anti-human	Biolegend	304042, 304024	1:200, 1:200-1:600	Flow cytometry	(AF700) B284831/ B306873	https://www.biolegend.com/ja-jp/quality/quality-control
CD45	BV510	30-F11	Rat, anti-mouse	Biolegend	103138	1:400-1:800	Flow cytometry	B251556/ B305756	https://www.biolegend.com/ja-jp/quality/quality-control
CD45RA	PE	HI100	Mouse, anti-human	BD	555489	1:100	Flow cytometry		https://www.bdbiosciences.com/en-us/products/reagents/flow-cytometry-reagents/research-reagents/quality-and-reproducibility
CD45RO	APC	UCHL1	Mouse, anti-human	BD	340438	1:100	Flow cytometry		https://www.bdbiosciences.com/en-us/products/reagents/flow-cytometry-reagents/research-reagents/quality-and-reproducibility
CD56	BV650, ECD	NCAM, N901	Mouse, anti-human	BD, Beckman Coulter	564057, A82943	1:200	Flow cytometry		https://www.bdbiosciences.com/en-us/products/reagents/flow-cytometry-reagents/research-reagents/quality-and-reproducibility<br>https://www.beckman.com/reagents/coulte
CD57	BV605	QA17A04	Mouse, anti-human	Biolegend	393303	1:200	Flow cytometry		https://www.biolegend.com/ja-jp/quality/quality-control
CD62L	PE-Cy7	DREG-56	Mouse, anti-human	Biolegend	304822	1:200	Flow cytometry		https://www.biolegend.com/ja-jp/quality/quality-control
CD123	BV605	6H6	Mouse, anti-human	Biolegend	306026	1:200	Flow cytometry		https://www.biolegend.com/ja-jp/quality/quality-control
CD135	PerCP-Cy5.5	BV10A4H2	Mouse, anti-human	Biolegend	313316	1:200	Flow cytometry		https://www.biolegend.com/ja-jp/quality/quality-control
CD137	PE	4B4-1	Mouse, anti-human	BD	555956	1:20	Flow cytometry		https://www.bdbiosciences.com/en-us/products/reagents/flow-cytometry-reagents/research-reagents/quality-and-reproducibility
CD197 (CCR7)	FITC	G043H7	Mouse, anti-human	BioLegend	353216	1:100	Flow cytometry		https://www.biolegend.com/ja-jp/quality/quality-control
HLA-A2	PE, BV650	BB7.2	Mouse, anti-human	BioLegend	343306, 343324	1:200	Flow cytometry	(PE) B279657 (BV650) B290341	https://www.biolegend.com/ja-jp/quality/quality-control
HLA-DR	AF700	L243	Mouse, anti-human	BioLegend	307626	1:200	Flow cytometry		https://www.biolegend.com/ja-jp/quality/quality-control
TCRβ	PE	H57-597	Mouse, anti-human	BioLegend	109208	1:200	Flow cytometry	B219253	https://www.biolegend.com/ja-jp/quality/quality-control
Ter119	PE-Cy5, BUV395	TER-119	Mouse, anti-human	Biolegend, BD	116210, 566206	1:600, 1:200	Flow cytometry	(PE-Cy5) B208715/B277 009 (BUV395) 7235927	https://www.biolegend.com/ja-jp/quality/quality-control<br>https://www.bdbiosciences.com/en-us/products/reagents/flow-cytometry-reagents/research-reagents/quality-and-reproducibility
7AAD				Biolegend		1:200	Flow cytometry	126M4105V	https://www.biolegend.com/ja-jp/quality/quality-control
DAPI				Invitrogren		1:50000	Flow cytometry	184346	https://www.thermofisher.com/no/en/home/life-science/antibodies/primary-antibodies.html
LIVE/DEAD™ Fixable Near-IR				Thermo Fisher Scientific	L10119	1:1000	Flow cytometry		https://www.thermofisher.com/no/en/home/life-science/antibodies/primary-antibodies.html
LIVE/DEAD™ Fixable Aqua Dead Cell Stain Kit				Thermo Fisher Scientific	L34957	1:200	Flow cytometry		https://www.thermofisher.com/no/en/home/life-science/antibodies/primary-antibodies.html
Cell Trace Violet				Thermo Fisher Scientific	C34557	1:3300	Flow cytometry		https://www.thermofisher.com/no/en/home/life-science/antibodies/primary-antibodies.html
5-(and-6)-Carboxyfluorescein Diacetate, Succinimidyl Ester				Thermo Fisher Scientific	C1157	1:2000	Flow cytometry		https://www.thermofisher.com/no/en/home/life-science/antibodies/primary-antibodies.html
Streptavidin	PE, APC			Thermo Fisher Scientific	S866, S868		Flow cytometry		https://www.thermofisher.com/no/en/home/life-science/antibodies/primary-antibodies.html
Purified Mouse Anti-Human IFN-γ		NIB42		BD	551221	1:500	ELISA		https://www.bdbiosciences.com/en-us/products/reagents/flow-cytometry-reagents/research-reagents/quality-and-reproducibility
Biotin Mouse Anti-Human IFN-γ		4S.B3		BD	554550	1:500	ELISA		https://www.bdbiosciences.com/en-us/products/reagents/flow-cytometry-reagents/research-reagents/quality-and-reproducibility
