# Supplementary material for: A T cell receptor targeting a recurrent driver mutation in FLT3 mediates elimination of primary human acute myeloid leukemia in vivo
Source: Nat Cancer. 2023 Oct 2;4(10):1474–90. doi: 10.1038/s43018-023-00642-8 (PMC10597840; doi:10.1038/s43018-023-00642-8)
Supplement: Supplementary file 2 — Reporting Summary [file 43018_2023_642_MOESM2_ESM.pdf]

nature portfolio

Corresponding author(s): Johanna Olweus

Last updated by author(s): Aug 23, 2023

Reporting Summary

Nature Portfolio wishes to improve the reproducibility of the work that we publish. This form provides structure for consistency and transparency in reporting. For further information on Nature Portfolio policies, see our Editorial Policies and the Editorial Policy Checklist.

Statistics

For all statistical analyses, confirm that the following items are present in the figure legend, table legend, main text, or Methods section.

n/a	Confirmed	
☐	☒	The exact sample size (*n*) for each experimental group/condition, given as a discrete number and unit of measurement
☐	☒	A statement on whether measurements were taken from distinct samples or whether the same sample was measured repeatedly
☐	☒	The statistical test(s) used AND whether they are one- or two-sided<br>*Only common tests should be described solely by name; describe more complex techniques in the Methods section.*
☒	☐	A description of all covariates tested
☐	☒	A description of any assumptions or corrections, such as tests of normality and adjustment for multiple comparisons
☐	☒	A full description of the statistical parameters including central tendency (e.g. means) or other basic estimates (e.g. regression coefficient) AND variation (e.g. standard deviation) or associated estimates of uncertainty (e.g. confidence intervals)
☐	☒	For null hypothesis testing, the test statistic (e.g. *F*, *t*, *r*) with confidence intervals, effect sizes, degrees of freedom and *P* value noted<br>*Give P values as exact values whenever suitable.*
☒	☐	For Bayesian analysis, information on the choice of priors and Markov chain Monte Carlo settings
☒	☐	For hierarchical and complex designs, identification of the appropriate level for tests and full reporting of outcomes
☒	☐	Estimates of effect sizes (e.g. Cohen's *d*, Pearson's *r*), indicating how they were calculated

Our web collection on statistics for biologists contains articles on many of the points above.

Software and code

Policy information about availability of computer code

Data collection

1. Flow cytometry was performed on BD LSR II Cytometer (BD Biosciences) and data was acquired with the help of BD FACSDIVA V8.0.1 software.
2. Cell sorting was performed with SH800 (Sony Biotechnology), BD FACSAria II (BD Biosciences) cell sorter and BD FACSAria Fusion cell sorter.
3. Immunoprecipitation- Mass spectrometry(IP-MS) data was acquired using an Ultimate 3000 nano-UHPLC system (Dionex, Sunnyvale, CA, USA) connected to a Q Exactive mass spectrometer (ThermoElectron, Bremen, Germany) equipped with a nano electrospray ion source.
4. Bioluminescence imaging of mice was performed with IVIS Spectrum in vivo imaging system (PerkinElmer).
5. Flow cytometry was performed on BD LSRFortessa (BD Biosciences).
6. Whole exome sequencing was performed using NextSeq.
7. Droplet digital PCR was performed using QX200 droplet generator (Bio-Rad) and QX200 droplet reader (Bio-Rad)

Data analysis

1. Flow cytometry data was analyzed with FlowJo version 9 and 10.
2.For the minigene design the peptide-MHC class I binding prediction algorithm NetMHC version 4.0 was used.
3. Numerical data was statistically analyzed and graphs were generated with the help of GraphPad Prism version 6, 7, 8 and 9 software.
4. Post-analysis of the LC-MS data was performed with Peaks DB (Bioinformatics Solutions Inc), using the Uniprot Homo sapiens database appended with the mutated protein in question.
5. Bioluminescence imaging was analyzed with Living image software version 4.5.2 (PerkinElmer)
6. Sequencing data was analyzed using the TruSight™ Myeloid Panel (Illumina, San Diego, CA, USA)
7. Curated human proteome databases UniProtKB/Swiss-Prot and Protein Data Bank were queried by employing ScanProsite tool (https://prosite.expasy.org/scanprosite/).
8. Whole exome sequencing reads were aligned to the human (GRCh37) genome reference using Burrows-Wheeler Aligner version 0.7.17 with default parameter settings. PCR duplicates were marked with biobambam version 2.0.87. Reads were subjected to indel realignment and

base quality score recalibration using GATK3 (version 3.8) and recalculation of MD/NM tags using SAMtools version 1.9. Mutation calling was performed through GenomonFisher (https://github.com/Genomon-Project/GenomonFisher). Called mutations were filtered and analyzed using R version 4.1.2.
9. Digital droplet PCR data was analyzed using QuantaSoft v1.5.38.1118 software (Bio-Rad).

For manuscripts utilizing custom algorithms or software that are central to the research but not yet described in published literature, software must be made available to editors and reviewers. We strongly encourage code deposition in a community repository (e.g. GitHub). See the Nature Portfolio guidelines for submitting code & software for further information.

Data

Policy information about availability of data

All manuscripts must include a data availability statement. This statement should provide the following information, where applicable:

- Accession codes, unique identifiers, or web links for publicly available datasets
- A description of any restrictions on data availability
- For clinical datasets or third party data, please ensure that the statement adheres to our policy

The data that support the findings of this study are included in the manuscript and in supplementary information. Additional datasets used in the study are: Uniprot Homo sapiens database, using Mascot v. 2.2.07 (www.matrixscience.com) ; curated human proteome databases UniProtKB/Swiss-Prot and PDB (Protein Data Bank) by ScanProsite tool (https://prosite.expasy.org/scanprosite/); and peptide-MHC class I binding prediction algorithm NetMHC version 4.0 (http://www.cbs.dtu.dk/services/NetMHC/); data from Papaemmanuil et al NEJM 2016 (PMID: 27276561, https://www.cbioportal.org); data from Morita et al Nat Commun 2020 (PMID: 33087716). Exome sequencing data have been deposited at the European Genome-phenome Archive (EGA), which is hosted by the EBI and the CRG, under accession number EGAS00001007467. Mass spectometry data have been deposited at the Proteomics Identification Database (PRIDE) under accession number PXD043908.

Human research participants

Policy information about studies involving human research participants and Sex and Gender in Research.

Reporting on sex and gender	Information regarding the patients' sex was collected and reported in Supplementary Table 2, main manuscript. This information was not considered during the design of the study but was part of the overall clinical information obtained at the hospital. No sex or gender based analysis was performed because all data generated from each patient is shown individually in the manuscript.
Population characteristics	Information about patient, disease and sample characteristics that was collected has been shown in the manuscript (Supplementary Table 2, Supplementary Table 3)
Recruitment	Approvals from the Institutional Review Board and the Regional Committee for Medical and Health Research Ethics (REC) South-East, Norway to use primary human diagnostic blood and bone marrow samples from adult patients in the study were obtained, as were informed written consent from the patients themselves. Patient samples were selected for testing after reviewing clinical diagnostic information regarding FLT3 TKD mutation and HLA-A2 status (positive/negative).<br>PB mononuclear cells (PBMCs) from healthy donor buffy coats were obtained from the blood bank of Oslo University Hospital, and PB or bone marrow mononuclear cells from leukemia patients were from biobanked, cryopreserved material (ethical approval numbers 2018/879, 2018/1246 and 2015/2357) or collected from Aarhus University Hospital with informed consent and ethical approval (1-45-70-88-21) and Karolinska University Hospital (2017/2085-31/2).
Ethics oversight	This study was approved by the Regional Committee for Medical and Health Research Ethics (REC) South-East Norway (2018/879, 2018/1246, and 2015/2357), the institutional Review Board and the Data Protection Officer, Oslo University Hospital, Swedish Ethical Review Authority, Stockholm (EPN 2017/2085-31/2), Ethical Committee in Central Denmark Region (1-45-70-88-21) and performed in accordance with the Declaration of Helsinki. Norwegian Food Safety Authority (application ID: 17500) and Stockholms Djurförsöksetiska nämnd (17978-2018) approved all animal experiments.

Note that full information on the approval of the study protocol must also be provided in the manuscript.

Field-specific reporting

Please select the one below that is the best fit for your research. If you are not sure, read the appropriate sections before making your selection.

☒ Life sciences ☐ Behavioural & social sciences ☐ Ecological, evolutionary & environmental sciences

For a reference copy of the document with all sections, see nature.com/documents/nr-reporting-summary-flat.pdf

Life sciences study design

All studies must disclose on these points even when the disclosure is negative.

Sample size	In the experiment with the xenograft leukemia cell line model we expected to see 50% decrease in the tumor bioluminescence signal in the

Sample size	treated animals compared to the control T-cell treated mice. In order to reach a p value of 0.05 with 95% power, we chose to have at least 3 mice per group.<br>For PDX models; no calculation of sample size was used, but sample size was determined based on expected treatment efficacy following the results observed on the xenograft model, where a minimum of 4 mice were included in each treatment group. This is also in line with other studies in the field from us and others (Ali, Giannakopoulou et al Nature Biotechnology 2022 and Jain, Zhao et al Nature 2023). Apart form this lower limit of number of mice, the number of patient cells available for transplantation was determining sample size of recipient mice. In PDX model 1, 6 mice were treated with 1G4 TCR T cells and 7 mice with FLT3D/Y TCR T cells. In PDX model 2, 4 mice in each treatment group was included. One mouse was left untreated and used for whole exome sequencing. In PDX model 3, four mice in each treatment group was included. In PDX model 4, at the start of the experiment 6 mice were engrafted with FLT3D/Y TCR and 1G4 TCR AML cultures each, and 4 mice with AML cultures without T cells, but some mice were excluded from analysis (see below).<br>For the in vitro experiments, at least three independent experiments were generally performed with different donors/patients. If less than three experiments were performed, a rationale is normally provided in the corresponding results section or figure legend. If less than three experiments were performed, no statistical calculations are applied.
Data exclusions	For all the PDX models, only mice that were able to be followed until the end of the experiment was included. No mice were excluded for PDX cohort 1, 2 and 3. For PDX model 4, a total of 5 mice were excluded. Three mice were excluded due to uncertainties during the intra-bone injections into the mice (one FLT3D/Y TCR and two 1G4 TCR) and two mice (one 1G4 TCR and one untreated) were excluded from the analysis because they were found dead before the last peripheral blood analysis was done and could therefore not be followed for the whole experiment. In the end, that left 5 FLT3D/Y TCR treated mice, 3 1G4 TCR treated mice and 3 untreated mice for the analysis.<br>No mice were excluded from the leukemia cell line model.<br><br>If 50% mean difference and standard deviation 17.5%, then we require n=3 per group.
Replication	Only one experiment was performed for the xenograft mouse model. This served as a proof of principle for FLT3TCR-mediated killing of mutation-expressing cells using a cell line not naturally expressing the target FLT3D835Y, which we introduced via retroviral transduction. The efficacy observed in this model provided the rationale for further testing of the TCR-T cell therapy in four disease-relevant mouse models, where NSG mice were engrafted with patient-derived acute myeloid leukemia cells.<br>Two independent patients were used to establish PDX models, and effective elimination of leukemic cells were observed using both patient models. Further, one of the patients (patient 1) was used in 3 different PDX models (model 2-4) and all showed similar results, indicating reproducibility. Finally, 2 independent experiments with PDX model 4 was done, and data presented is pooled from both with n = 4 and n = 7 respectively. These mice were distributed among treatment groups as followed:<br>Exp nr. 1, 2 FLT3D/Y TCR treated, 1 1G4 TCR treated and 1 untreated<br>Exp nr 2, 3 FLT3D/Y TCR treated, 2 1G4 TCR treated and 2 untreated<br>For all the in vitro data at least three independent experiments were generally performed, showing successful replication of data, as described in detail in figure legends.
Randomization	In the experiment with the xenograft leukemia cell line model, mice injected with tumor were randomly assigned in different groups one day before T cell therapy. There was no difference in the tumor BLI signal between experimental groups before start of therapy (data shown in the manuscript).<br>For experiments using PDX model 1-3, mice mice were assigned to different treatment groups based upon their engraftment levels in blood to ensure that both groups had similar mean engraftment levels. For PDX model 2, mice were also allocated based on sex so that there were equal number of male and female mice in each treatment group. For experiments using PDX model 4, co-cultures were injected randomly into mice. For the in vitro experiments, randomization was not performed as it was not applicable and is not generally performed in the field.
Blinding	Investigators were not blinded to allocation of mice during experiments and analysis. Blinding would be impractical due to the limitations related to manpower, and because of the potential alloreactivity that needed to be closely assessed. Due to the nature of the other in vitro experiments, blinding was not possible and is not generally performed in the field as the data acquisition is quantitative (flow cytometry or MS) rather than qualitative and therefore less influenced by observer bias.

Reporting for specific materials, systems and methods

We require information from authors about some types of materials, experimental systems and methods used in many studies. Here, indicate whether each material, system or method listed is relevant to your study. If you are not sure if a list item applies to your research, read the appropriate section before selecting a response.

Materials & experimental systems

n/a	Involved in the study	
☐	☒	Antibodies
☐	☒	Eukaryotic cell lines
☒	☐	Palaeontology and archaeology
☐	☒	Animals and other organisms
☒	☐	Clinical data
☐	☒	Dual use research of concern

Methods

n/a	Involved in the study	
☒	☐	ChIP-seq
☐	☒	Flow cytometry
☒	☐	MRI-based neuroimaging

Antibodies

Antibodies used	The following fluorescently conjugated anti-human and anti-mouse antibodies were acquired from BD Biosciences or BioLegend

Antibodies used

unless otherwise specified: Anti-human CD3 (SK7, UCH-T1, HIT3α), -CD4 (OKT4, RPA-T4, SK3), -CD8a (HIT8a, RPA-T8), -CD11b (ICRF44), -CD13 (WM15), -CD14 (HCD14, M5E2), -CD16 (3G8, NKP15), -CD19 (HIB19, SJ25c1, 4G7), -CD20 (2H7), -CD33 (P67.6, VM-53), -CD34 (561, 581, 8G12), -CD38 (HB-7), -CD45 (30-F11, H130), -CD45RA (HI100), –CD45RO (UCHL1), -CD56 (NCAM, N901 Beckman Coulter), -CD57 (QA17A04), -CD62L (DREG-56), -CD123 (6H6), -CD135 (BV10A4H2), -CD137 (4B4-1), -CD197 (G043H7), -HLA-A2 (BB7.2), -HLA-DR (L243), anti-mouse CD45 (30-F11) and -Ter199 (TER119). Anti-mouse TCR-β chain (H57-597) was used to determine transduction efficiency of the TCR1G4 or TCRFLT3D/Y in human cells and monitor transduced T cells used for in vivo treatment in mice. Live/Dead Fixable Near-IR Dead Cell Stain kit and Aqua Dead Cell Stain kit (Life Technologies), 7AAD (Sigma Aldrich) or DAPI (Thermofisher) was used to exclude dead cells in all flow cytometry experiments. The following antibodies for ELISA were acquired from BD Pharmingen or R&D systems: mouse anti-human IFNγ capure antibody (NIB42) and Biotin Mouse Anti-Human IFN-γ detection antibody (4S.B3). Detailed information is provided in Supplementary Table 10 and below:

CD3 PerCP-Cy5.5, APC-Cy7, BB515, SK7, UCH-T1, HIT3a Mouse, anti-human Biolegend, Biolegend, BD 344808, 300426, 565100 1:200, 1:200, 1:100-1:200 Flow cytometry (BB515) 7158825/
9021562/
9205788/
0077790
CD4 BUV395, PE-Cy5, BV711 RPA-T4, OKT4 Mouse, anti-human BD, Biolegend, Biolegend 564724, 300512, 317440 1:300, 1;300, 1:200 Flow cytometry (BUV395) 35962, (PE-Cy7) B269744/
B288879
CD8a AF700, BV421, APC-Cy7 HIT8a, RPA-T8 Mouse, anti-human Biolegend 300920, 301036, 301016 1:200, 1:200, 1:80-1:300 Flow cytometry (APC-Cy7) B274260/ B300873
CD11b FITC ICRF44 Mouse, anti-human Biolegend 301330 1:200 Flow cytometry
CD13 BV711 WM15 Mouse, anti-human Biolegend 301722 1:200 Flow cytometry
CD14 FITC, BV711, PE-Cy7 HCD14, M5E2 Mouse, anti-human Biolegend 325604, 301838, 301820 1:200 Flow cytometry
CD16 FITC 3G8, NKP15 Mouse, anti-human Biolegend, BD 302006, 335035 1:200, 1:100 Flow cytometry
CD19 BV785, BV421, FITC HIB19, SJ25c1, 4G7), Mouse, anti-human Biolegend, Biolegend, BD 302240, 363018, 345776 1:200, 1:200-1:300, 1:100 Flow cytometry (BV421) B237452/B313190
CD20 BV785 2H7 Mouse, anti-human Biolegend 302356 1:200 Flow cytometry
CD33 APC-Cy7, BV785 P67.6, VM-53 Mouse, anti-human Biolegend 366614, 303428 1:200 Flow cytometry (BV785) B252804/
B260381/
B297609
CD34 BV785, AF647, APC 561, 581, 8G12 Mouse, anti-human Biolegend, Biolegend, BD 343626, 343508, 345804 1:200 Flow cytometry (APC) 7348707
CD38 PE-Cy7 HB-7 Mouse, anti-human Biolegend 356608 1:200 Flow cytometry
CD45 BV605, AF700 H130 Mouse, anti-human Biolegend 304042, 304024 1:200, 1:200-1:600 Flow cytometry (AF700) B284831/
B306873
CD45 BV510 30-F11 Rat, anti-mouse Biolegend 103138 1:400-1:800 Flow cytometry B251556/
B305756
CD45RA PE HI100 Mouse, anti-human BD 555489 1:100 Flow cytometry
CD45RO APC UCHL1 Mouse, anti-human BD 340438 1:100 Flow cytometry
CD56 BV650, ECD NCAM, N901 Mouse, anti-human BD, Beckman Coulter 564057, A82943 1:200 Flow cytometry
CD57 BV605 QA17A04 Mouse, anti-human Biolegend 393303 1:200 Flow cytometry
CD62L PE-Cy7 DREG-56 Mouse, anti-human Biolegend 304822 1:200 Flow cytometry
CD123 BV605 6H6 Mouse, anti-human Biolegend 306026 1:200 Flow cytometry
CD135 PerCP-Cy5.5 BV10A4H2 Mouse, anti-human Biolegend 313316 1:200 Flow cytometry
CD137 PE 4B4-1 Mouse, anti-human BD 555956 1:20 Flow cytometry
CD197 (CCR7) FITC G043H7 Mouse, anti-human BioLegend 353216 1:100 Flow cytometry
HLA-A2 PE, BV650 BB7.2 Mouse, anti-human BioLegend 343305, 343306, 343324 1:100, 1:200 Flow cytometry (PE) B279657 (BV650) B290341
HLA-DR AF700 L243 Mouse, anti-human BioLegend 307626 1:200 Flow cytometry
TCRβ PE H57-597 Mouse, anti-human BioLegend 109208 1:200 Flow cytometry B219253
Ter119 PE-Cy5, BUV395 TER-119 Mouse, anti-human Biolegend, BD 116210, 566206 1:600, 1:200 Flow cytometry (PE-Cy5) B208715/B277009 (BUV395) 7235927
7AAD Biolegend 1:200 Flow cytometry 126M4105V
DAPI Invitrogren 1:50000 Flow cytometry 184346
LIVE/DEAD™ Fixable Near-IR Thermo Fisher Scientific L10119 1:1000 Flow cytometry
LIVE/DEAD™ Fixable Aqua Dead Cell Stain Kit Thermo Fisher Scientific L34957 1:200 Flow cytometry
Cell Trace Violet Thermo Fisher Scientific C34557 1:3300 Flow cytometry
5-(and-6)-Carboxyfluorescein Diacetate, Succinimidyl Ester Thermo Fisher Scientific C1157 1:2000 Flow cytometry
Streptavidin PE, APC Thermo Fisher Scientific S866, S868 Flow cytometry
Purified Mouse Anti-Human IFN-γ NIB42 BD 551221 1:500 ELISA
Biotin Mouse Anti-Human IFN-γ 4S.B3 BD 554550 1:500 ELISA

Validation

All antibodies used in the study are available commercially and have been validated by commercial vendors for use in research or diagnostics. Furthermore, all antibodies used have been individually titrated prior to use to identify their optimal concentration in the required application. Detailed information is provided in Supplementary Table 10.

Eukaryotic cell lines

Policy information about cell lines and Sex and Gender in Research

Cell line source(s)

The following cell lines were all authenticated and obtained from American Type Culture Collection (ATCC) or German Collection of Microorganisms and Cell Cultures (DSMZ) or were kindly gifted (giver in parenthesis): NALM-6 and BV173 (Dr.

June Myklebust), RS4;11 (DSMZ), T2 (ATCC), RD (ATCC), U-2 OS, U-87 MG, ML-2, FM6 and COLO 668 (Dr. Fridtjof Lund-Johansen), UT-7 (DSMZ), HeLa (Dr. Andreas Brech), HaCaT (Dr. Frode Jahnsen), MCF7 (Dr. Matthias Leisegang), K562 (ATCC), EA.hy926 (ATCC), Daoy (ATCC), HCT-116 (DSMZ), CHP-212 (DSMZ), OCI-M2 (DSMZ), HEK 293 (ATCC), Hep G2 (ATCC), MV-4-11 (DSMZ), EoL-1 (DSMZ), MOLM-13 (DSMZ), Caco-2 (Dr. Ragnhild A. Lothe), HEL 92.1.7 (ATCC), HLA class-I deficient B721.221 cells were obtained from FRED HUTCH Research Cell Bank, UT-7, Phoenix-AMPHO (ATCC). EBV-LCL cell line was previously generated in-house by immortalizing human PBMC's from HLA-A2 positive and negative donors with Epstein-Barr viral supernatants. Sex or gender of the origin of cell lines was not taken into consideration and cell lines were selected based on HLA and tissue origin.All cell lines were cryopreserved in aliquots labeled according to passage and only low passage cell lines were used to start fresh cultures

Authentication

Authenticated cell lines (STR DNA profiling) were purchased from ATCC or DSMZ: RS4;11, T2, RD, K562, EA.hy926, Daoy, HCT-116, CHP-212, HEL, 92.1.7, B721.221, MV4-11, EoL-1, MOLM-13, Pheonix AMPHO, and cryopreserved aliquots labeled according to passage. Only low passages (1-4 passages) were used to start cultures. The identity of the passage used (5 or higher) experimentally of the cell lines NALM-6, BV173, U-2 OS, FM6, HeLa, HaCaT, MCF7, COLO 668, U-87MG , ML-2, OCI-M2, HEK 293, Caco-2, UT-7, Hep G2 was ascertained by short tandem repeat DNA profiling, a service provided by Labcorp DNA identification LAb, NC, USA (formely Genetica, https://celllineauthentication.com/). In-house immortalized EBV-LCL cells were regularly tested for CD20 or CD19 staining to confirm their B cell origin.

Mycoplasma contamination

Cells were tested regularly for mycoplasma contamination and were confirmed negative before experimental use

Commonly misidentified lines
(See ICLAC register)

No commonly misidentified cell lines were used in the study.

Animals and other research organisms

Policy information about studies involving animals; ARRIVE guidelines recommended for reporting animal research, and Sex and Gender in Research

Laboratory animals

In PDX model 1, NSG-SGM3 mice (NOD.Cg-Prkdcscid Il2rgtm1Wjl Tg(CMV-IL3,CSF2,KITLG)1Eav/MloySzJ) stably engrafted with primary FLT3D/Y HLA-A2pos AML patient cells at 5 weeks of age were obtained from the Jackson Laboratory (Stock ID J000106565). In PDX model 2, NSG-SGM3 mice (NOD.Cg-Prkdcscid Il2rgtm1Wjl Tg(CMV-IL3,CSF2,KITLG)1Eav/MloySzJ, obtained from Jackson Laboratory; stock 013062) were engrafted with FLT3D/Y HLA-A2pos AML patient cells at 9 weeks of age. In PDX model 3, NSG mice (NOD.Cg-Prkdcscid Il2rgtm1Wjl/SzJ, obtained from Jackson Laboratory; stock 005557) were engrafted at 12-13 weeks of age with FLT3D/Y HLA-A2pos AML patient cells previously engrafted into NSG mice at 8-13 weeks of age. In PDX model 4, NOG-hIL2 mice (NOD.Cg-Prkdcscid Il2rgtm1Sug Tg(CMV-IL2)4-2Jic/JicTac, obtained from Taconic) engrafted with FLT3D/Y HLA-A2pos AML patient cells at 14 weeks of age were used as donors for co-cultures. Co-cultures were transplanted into NSG mice (NOD.Cg-Prkdcscid Il2rgtm1Wjl/SzJ, obtained from Jackson Laboratory; stock 005557) at 8-13 weeks of age.
All mice were housed 2-5 mice per cage in IVC-Mouse GM 500 cages with a light cycle of 4 a.m – 4 p.m (patient 7) or 6 a.m – 6 p.m (patient 1), 21°C and 50% humidity. The maximal tumour burden permitted by the ethics committee/institutional review board was defined by the impact on the animal's health. Briefly, mice engrafted with leukemic cells were continuously monitored for signs of poor health according to Oslo University’s and Karolinska Institutet’s health assessment where animals receiving a score above 0.4 points were terminated. In our experiments, no animal exceeded the humane endpoint.
For establishing xenograft leukemia cell line model, 8- to 10-weeks-old female NOD.Cg-Prkdcscid Il2rgtm1Wjl/SzJ (nsg) mice were used. All mice were housed 6 mice per cage in Eurostandard type III cages (macrolone) with a light cycle of 7 a.m – 7 p.m, 22 ± 1C and 62 ± 5% humidity. Maximum tumor burden for leukemia is indirectly measured. Mice were observed for clinical signs of tumor spread and were sacrificed if they developed >20% weight loss, hunched posture, ruffled fur, limb paralysis or enlarged spleens (distended abdomens, enlarged and palpable spleens). Maximum tumor burden was not exceeded. Experiments were terminated 2 months after T-cell injection to avoid graft-versus-host disease, and surviving mice in treated groups were humanely sacrificed.

Wild animals

No wild animals were used.

Reporting on sex

For the xenograft leukemia cell line model only female mice were use.
In PDX model 2, both male and female mice were used and equally distributed between the treatment groups. For all other PDX models, only female mice were used.

Field-collected samples

No field-collected samples were used.

Ethics oversight

Experiments were performed according to the guidelines and obtained permissions from the ethics committees at Stockholm Norra Djurförsöksetisks Nämd (17978-2018) and Norwegian Food Safety Authority (17500). For the cell line xenograft model, mice were observed for clinical signs of tumor spread and were sacrificed if they developed >20% weight loss, hunchedposture, ruffled fur, limb paralysis or enlarged spleens (distended abdomens, enlarged and palpable spleens). Experiments were terminated 2 months after T-cell injection to avoid graft-versus-host disease, and surviving mice in treated groups were humanely sacrificed. The maximum tumor burden was not exceeded in any mice. For the PDX models the maximal tumour burden permitted was defined by the impact on the animal's health. Briefly, mice engrafted with leukemic cells were continuously monitored for signs of poor health according Karolinska Institutet’s health assessment where animals receiving a score above 0.4 points were terminated. In our experiments, no animal exceeded the humane endpoint.

Note that full information on the approval of the study protocol must also be provided in the manuscript.

Dual use research of concern

Policy information about dual use research of concern

Hazards

Could the accidental, deliberate or reckless misuse of agents or technologies generated in the work, or the application of information presented in the manuscript, pose a threat to:

No	Yes	
☒	☐	Public health
☒	☐	National security
☒	☐	Crops and/or livestock
☒	☐	Ecosystems
☒	☐	Any other significant area

Experiments of concern

Does the work involve any of these experiments of concern:

No	Yes	
☒	☐	Demonstrate how to render a vaccine ineffective
☒	☐	Confer resistance to therapeutically useful antibiotics or antiviral agents
☒	☐	Enhance the virulence of a pathogen or render a nonpathogen virulent
☒	☐	Increase transmissibility of a pathogen
☒	☐	Alter the host range of a pathogen
☒	☐	Enable evasion of diagnostic/detection modalities
☒	☐	Enable the weaponization of a biological agent or toxin
☒	☐	Any other potentially harmful combination of experiments and agents

Flow Cytometry

Plots

Confirm that:

☒ The axis labels state the marker and fluorochrome used (e.g. CD4-FITC).

☒ The axis scales are clearly visible. Include numbers along axes only for bottom left plot of group (a 'group' is an analysis of identical markers).

☐ All plots are contour plots with outliers or pseudocolor plots.

☒ A numerical value for number of cells or percentage (with statistics) is provided.

Methodology

Sample preparation

Samples analyzed by flow cytometry contained mononuclear cells in suspension isolated from buffy coats by lymphoprep density gradient centrifugation that were cultured with standard media as detailed in the manuscript. Patient blood or bone marrow samples were harvested for diagnostic purposes and mononuclear cells were isolated by lymphoprep density gradient centrifugation. Samples were then cryopreserved and stored in liquid nitrogen in designated cell biobanks in our institutions before use in the experiments as detailed in the methods. Cell lines utilized were cultured for variable amounts of time in recommended media before test in the experiments.
Blood samples and bone marrow were harvested from murine xenograft leukemia cell line models. RBC lysis was performed by ACK lysis buffer, followed by washing and surface or intracellular staining with different anti-bodies for flow cytometry analysis.
For the PDX mice, peripheral blood (tail vein or cardiac puncture) was harvested and processed with 1:1 Dextran followed by red blood cell lysis with ammonium-chloride and processed into single cell suspension in PBS supplemented with 1-5 % fetal calf serum and 2mM EDTA. Bone marrow (tibia, femur, cristae from both hind legs) from PDX mice was harvested from PDX mice and crushed with mortal and pestle and processed into single cell suspension in PBS supplemented with 1-5 % fetal calf serum and 2mM EDTA. Spleen was processed into single cell suspension in PBS supplemented with 1-5 % fetal calf serum and 2mM EDTA. All samples were includated with both anti mouse and anti human FcR-block before staining with monoclonal antibodies.

Instrument

BD LSR II (BD Biosciences) equipped with high throughput sampler (HTS), BD LRSFortessa (BD Biosciences). Cell sorting was performed with SH800 (Sony Biotechnology), BD FACSAria II (BD Biosciences) cell sorter and BD FACSAria Fusion.

Software

For data collection for all experiments, BD FACSDiva V8.0.1 was used. For data analysis FlowJo version 9 and 10 was used.

Cell population abundance

Tumor cell lines that were transduced to express the FLT3 D835Y mutation and GFP and firefly luciferase were purified by FACS with a purity > 95%.
For the PDX models purity sorts were done with >95 % purity of relevant populations. Purity was determined by sorting of >100 cells for each relevant population and re-analysis of the sorted cells.

Gating strategy

For all flow cytometry experiments FSC-A/SSC-A was used for gating mononuclear cells. Doublets were excluded. 7AAD, DAPI Live/dead fixable near-IR -or Live/dead Aqua positive cells were gated out to exclude non-viable cells.

For pMHC multimer staining: From the live cell gate, CD8+ events were gated and subsequently, pMHC multimer+ events were identified as double positive for PE and APC conjugated pMHC multimers.

For flow cytometry based cytotoxocity assays and T cell activation assays, tumor cell lines and transduced T cells were stained with surface anti-bodies and were gated as detailed in the methods and extended data supplement. Primary patient samples were also stained with surface antibodies for the presence of CD3- CD19- CD20- myeloid cells. In this case, effector cells in the same co-culture well were pre-labeled with CellTrace Violet (CTV, Life Technologies) to distinguish from live target cells. Myeloid cells (CD3- CD19- CD20-), mature B and T cells, and CD34+lin- cells were gated and overlaid to be visualized as T-Distributed Stochastic Neighbor Embedding (tSNE) plots. CountBright Absolute Counting Beads were utilized to acquire equal numbers of events in each tested well.

To analyze presence of transduced T cells in blood and bone marrow of the xenograft leukemia cell line model, single live cells are gated as described above. Total leukocytes were defined as antigen positive for both human and mouse CD45. From CD45+ gate, human TCR transduced T cells were identified as anti-human CD3+, CD8+ and anti-mouse TCR-β+. A murine constant part was introduced into the TCR-β chain to serve as a reporter for TCR transduction.

To determine leukemic burden in PDX mice, viable (7AAD or DAPI negative) single (SSC-W vs SS-H) human and mouse leukocytes were separated based on mTer119- cells staining positive for either mouse or human CD45. Further, AML cells were determined as hCD45+CD3-CD19-CD33+. For PDX model 2, two distinct CD33+ populations were identified as CD34+ or CD34-. To track the infused T cells, the hCD45+CD3+ cells were further gated on CD4+ or CD8a+ and mTCRb+.

☒ Tick this box to confirm that a figure exemplifying the gating strategy is provided in the Supplementary Information.
